# Exercise motivation is stable yet mobile: a three-wave latent transition study of psychological-need predictors and subsequent exercise

**DOI:** 10.3389/fpsyg.2026.1967142

**Published:** 2026-10-29

**Authors:** Chenhui Zhu, Qingyang Huang, Weikun Liu

**Affiliations:** School of Sports and Health, Yancheng Teachers University, Jiangsu, China

**Keywords:** exercise regulation, latent transition analysis, leisure-time exercise, motivational profiles, psychological need frustration, psychological need satisfaction, self-determination theory

## Abstract

**Background:**

Most person-centered research on exercise motivation has identified static profiles, leaving unclear how motivational configurations change over time, what psychological experiences are associated with those changes, and whether transitions predict subsequent exercise. This study examined the short-term mobility of exercise-regulation profiles, psychological-need predictors of specific transitions, and their prospective associations with later leisure-time exercise.

**Methods:**

A three-wave prospective study followed 1,780 students from six Chinese colleges at approximately 4-week intervals. Exercise regulation, exercise-specific psychological need satisfaction and frustration, and leisure-time exercise were assessed at each wave. Continuous-indicator latent transition analysis identified longitudinal motivational profiles and transition probabilities. Posterior-probability-weighted models examined origin-wave need satisfaction and frustration as predictors of eligible transitions and tested whether first-interval transitions were associated with final-wave exercise after prior exercise was considered.

**Results:**

Six profiles were retained: amotivated–controlled, low-regulation, mixed intrinsic–controlled, identified-dominant, intrinsic-dominant, and high autonomous–introjected. Overall profile stability was substantial but incomplete, with 76.9 and 73.8% of posterior transition mass remaining on the stability diagonal across the two intervals. The largest off-diagonal transition was from low regulation to high autonomous–introjected motivation (18.0 and 20.1%). After Holm correction, greater need satisfaction was associated with lower odds of moving from intrinsic-dominant or high autonomous–introjected motivation to low regulation (odds ratios = 0.47–0.49), whereas greater need frustration was associated with lower odds of moving from low regulation to identified- or intrinsic-dominant profiles (odds ratios = 0.41–0.62). In models controlling baseline exercise, movement from amotivated–controlled or low regulation to high autonomous–introjected motivation was associated with greater subsequent exercise, whereas several transitions toward less internalized configurations were associated with lower exercise. After additional adjustment for exercise measured at the transition endpoint, only the low-regulation-to-high autonomous–introjected transition remained significant.

**Conclusion:**

Exercise motivation was substantially stable yet meaningfully mobile over short periods. Psychological-need experiences were associated with specific, origin-dependent motivational transitions, and selected transitions prospectively differentiated later exercise behavior. The findings support a dynamic and state-dependent view of exercise motivation and suggest that the psychological conditions relevant to maintaining internalized motivation may differ from those involved in moving out of low regulation.

## 1 Introduction

Sustaining exercise during college depends not only on whether students are motivated, but also on why they exercise. Organismic Integration Theory, a component of Self-Determination Theory, distinguishes exercise regulations by their degree of internalization and self-endorsement (Deci and Ryan, 2000; Ryan and Deci, 2017). Amotivation reflects little intention or meaningful reason to exercise. External regulation involves rewards, demands, or interpersonal pressure, whereas introjected regulation involves internal pressure such as guilt, obligation, or contingent self-worth. Identified regulation reflects personally valuing exercise and its outcomes. Integrated regulation represents a more fully internalized form of extrinsic motivation in which the value of the behavior has been assimilated with the person’s broader values, identity, and sense of self. Intrinsic regulation reflects exercising for enjoyment or inherent satisfaction. Identified and integrated regulation, together with intrinsic regulation, are comparatively autonomous, whereas external and introjected regulations are comparatively controlled. Exercise research generally links identified and intrinsic regulations with greater participation and persistence, while the role of introjected regulation is more conditional (Teixeira et al., 2012). Importantly, these regulations can coexist. A student may enjoy and value exercise while also feeling guilty or pressured, making the configuration of motives potentially more informative than any regulation considered alone.

Most research has examined exercise regulations separately or combined them into broad autonomous- and controlled-motivation scores. Such variable-centered approaches estimate average associations but can conceal meaningful within-person combinations. Person-centered methods instead identify subgroups from individuals’ complete motivational patterns. Lindwall et al. (2017), for example, identified six exercise-motivation profiles across two adult samples, including low-motivation, self-determined, and self-determined-with-high-introjection configurations; participants in more self-determined profiles also reported greater psychological-need satisfaction. These findings show that autonomous and controlled reasons are not necessarily opposites within individuals. They also caution against assuming a universal profile structure: strongly autonomous and weakly internalized configurations may recur, but the number and form of intermediate or mixed profiles can vary across populations and contexts (Emm-Collison et al., 2020; Lindwall et al., 2017).

Recent studies confirm substantial motivational heterogeneity among Chinese college students. Gao et al. (2025) identified five profiles, including high-autonomy and high-binding configurations, and found profile differences in academic stress and physical education engagement. Using the five regulations of the Chinese Behavioral Regulation in Exercise Questionnaire-2, Zheng et al. (2026) identified four profiles and reported greater exercise adherence in high-autonomous and intrinsic-dominant profiles than in amotivated–controlled and identified-dominant profiles. These studies establish the relevance of a person-centered approach in Chinese higher education, but both were cross-sectional. They could not determine whether students retained the same motivational configuration, moved toward or away from more internalized regulation, or whether such movement predicted later exercise.

This limitation is theoretically important because Organismic Integration Theory conceptualizes internalization as a process rather than a fixed trait. Latent transition analysis can distinguish population-level prevalence from individual-level mobility by estimating probabilities of remaining in or moving between profiles. In one of the few directly relevant studies, Emm-Collison et al. (2020) followed 2,555 British parents across three assessments over 5 years. Six exercise-motivation profiles emerged; more autonomous profiles were comparatively enduring and were associated with higher accelerometer-measured moderate-to-vigorous physical activity and lower body mass index. Ruzella et al. (2026) more recently examined transitions in exercise motive-content profiles among low-active middle-aged adults, but those profiles represented motives such as fitness and psychological wellbeing rather than the behavioral regulations specified by Organismic Integration Theory. Longitudinal person-centered evidence therefore remains limited, concentrated in Western adult samples, and based on constructs that are not always interchangeable. This concentration matters for both cultural and developmental reasons. Self-Determination Theory proposes that autonomy, competence, and relatedness are broadly relevant across cultures, but it also recognizes that cultural practices, social expectations, and interpersonal contexts can shape how values and behavioral regulations are internalized and expressed. Cross-cultural evidence accordingly supports the importance of autonomous functioning in both Western and non-Western settings while also documenting variation in the social pathways through which motivational processes operate (Chirkov et al., 2003; Nalipay et al., 2020). The present college-aged sample also occupies emerging adulthood, a developmental period characterized by increasing responsibility for self-directed choices, identity development, and changing social influences (Ratelle and Guay, 2023). Exercise research further suggests that some associations among psychological needs, motivational regulation, and behavior can vary with age (Weman Josefsson et al., 2018). Profile structure and mobility observed among Western parents or middle-aged adults therefore should not automatically be assumed to characterize Chinese college students. To our knowledge, no published study has integrated three-wave exercise-regulation profiles, psychological-need predictors of profile transitions, and later exercise among Chinese college students.

Understanding whether motivational profiles are stable or mobile also has practical implications for intervention. Stability indicates whether a motivational configuration is likely to persist without substantial change: a stable, comparatively internalized profile may primarily require maintenance, whereas a stable low-regulation or amotivated configuration may indicate a more persistent barrier to exercise engagement. Conversely, profiles that frequently improve or deteriorate can reveal potential windows for supporting internalization or preventing motivational decline. Transition evidence can therefore distinguish different intervention objectives—preserving already-internalized motivation, helping students move out of weak or controlled regulation, and preventing deterioration from more internalized configurations—rather than assuming that the same motivational strategy is appropriate for everyone. Recent work in motivational design outside the exercise domain similarly argues for “design for internalisation,” in which support is calibrated to individuals’ existing motivational configurations and seeks to scaffold more sustainable forms of regulation over time (Bennett and Mekler, 2024; Bennett et al., 2025). Although that work concerns technology use, the broader intervention principle is directly relevant here: effective support may depend on both a person’s motivational starting state and the kinds of motivational change that are realistically observed.

Identifying transitions, however, does not explain why they occur. Basic Psychological Needs Theory proposes that internalization and optimal functioning are supported by satisfaction of the needs for autonomy, competence, and relatedness (Deci and Ryan, 2000; Ryan and Deci, 2017). In exercise, these experiences involve feeling that participation is self-endorsed, that one is capable, and that one is meaningfully connected with others. Need frustration reflects active experiences of coercion, inadequacy, failure, or exclusion and is not simply the absence of satisfaction (Chen et al., 2015; Vansteenkiste et al., 2020). Need satisfaction should make exercise more likely to be experienced as chosen, meaningful, manageable, and socially supported, thereby facilitating internalization; need frustration may preserve pressure-based regulation, impede internalization, or foster disengagement. Exercise research also indicates that satisfaction and frustration can coexist and show differentiated relations with behavioral regulations (Rodrigues et al., 2021).

These need effects may be state-dependent. Higher need satisfaction could protect students with strongly internalized motivation from deteriorating toward low regulation, yet help students beginning in a controlled or weakly regulated profile move toward identified or intrinsic regulation. Conversely, need frustration could destabilize an autonomous configuration or prevent a low-regulation student from developing more internalized reasons for exercise. An origin-specific transition framework can test these possibilities directly. The relevant question is not only whether psychological needs correlate with motivation on average, but which origin–destination movements they make more or less likely. Importantly, person-centered regulation profiles are multidimensional configurations rather than necessarily ordered positions on a single autonomy continuum. Profiles can combine comparatively autonomous and controlled regulations, such that two destinations that appear generally “more internalized” may nevertheless represent substantively different motivational changes. We therefore conceptualized psychological-need effects as origin- and destination-specific rather than assuming that all transitions could be collapsed into a single upward-versus-downward dimension.

The behavioral meaning of motivational mobility is equally important. Cross-sectional profile differences show that motivational configurations are associated with exercise, but they do not establish whether changing configurations forecasts later behavior. A stronger prospective test asks whether movement toward or away from a comparatively internalized profile predicts subsequent leisure-time exercise after earlier exercise is considered. Movement from low, controlled, or amotivated regulation toward identified or intrinsic regulation should generally predict greater subsequent leisure-time exercise, whereas movement toward less internalized regulation should predict lower subsequent exercise. Transitions between two comparatively internalized profiles may have smaller or more nuanced consequences because autonomous and introjected motives can coexist. Linking need experiences to subsequent transitions and early transitions to later exercise can therefore show whether motivational mobility is both psychologically explicable and behaviorally consequential. These are connected prospective associations, not a formal test of causal mediation.

Chinese college students provide an informative context for this investigation. College increases personal responsibility for schedules, leisure choices, and health behavior, while exercise remains shaped by academic demands, facilities, guidance, social relationships, and campus opportunities. A systematic review identified academic time pressure, limited facilities, and insufficient professional guidance among the barriers to exercise in this population (Pan et al., 2022). Such conditions may foster the coexistence of autonomous and controlled motives and create opportunities for short-term motivational change. Examining students across bachelor’s-degree and vocational colleges also broadens the institutional context beyond a single conventional university, although it does not make the sample nationally representative.

The present study followed Chinese college students from six colleges across three assessment occasions: baseline, designated Time 1 (T1); approximately 4 weeks later, designated Time 2 (T2); and approximately 4 weeks after T2, designated Time 3 (T3). It first identified longitudinal profiles based on amotivation, external regulation, introjected regulation, identified regulation, and intrinsic regulation. It then estimated profile stability and movement from T1 to T2 and from T2 to T3, tested exercise-specific need satisfaction and need frustration as origin-wave predictors of eligible transitions relative to stability in the same profile, and examined whether transitions from T1 to T2 prospectively predicted leisure-time exercise at T3 after baseline exercise was controlled. The study thereby integrates Organismic Integration Theory and Basic Psychological Needs Theory within a dynamic internalization framework, examines complete motivational configurations rather than isolated regulations, and evaluates whether short-term motivational mobility is both psychologically explicable and behaviorally consequential. By linking profile persistence and change with their psychological-need correlates and subsequent behavioral implications, the study moves beyond identifying who occupies different motivational profiles to asking which motivational configurations persist, which changes occur, what experiences are associated with those changes, and which transitions matter for later exercise. This distinction is important for developing a more state-sensitive account of exercise motivation and, ultimately, for informing interventions that differentiate between maintaining internalized motivation, facilitating internalization, and preventing motivational deterioration. Three research questions guided the study. The first research question (RQ1) asked what longitudinal exercise-regulation profile structure best represented Chinese college students across T1, T2, and T3 and what the probabilities were of remaining in or moving between those profiles. Because the number and form of latent profiles and their relative stability are data-dependent features of mixture models, RQ1 was treated as exploratory rather than confirmatory; prior theory and research guided interpretation but were not used to specify an *a priori* profile count or quantitative stability threshold. The second research question (RQ2) asked whether exercise-specific need satisfaction and need frustration would prospectively predict selected profile transitions relative to stability in the same origin profile. The third research question (RQ3) asked whether transitions from T1 to T2 toward or away from comparatively autonomous exercise regulation would be prospectively associated with leisure-time exercise at T3 after exercise reported at baseline was controlled.

Three directional hypotheses were advanced for the theory-driven prospective components of RQ2 and RQ3. The first hypothesis (H1) predicted that greater need satisfaction would show profile-specific associations consistent with preserving or developing internalized motivation: specifically, lower likelihood of moving from comparatively internalized configurations toward low-regulation, controlled, or amotivated configurations relative to remaining in the same origin profile, and greater likelihood of movement from weakly regulated configurations toward profiles characterized by stronger identified or intrinsic regulation. The second hypothesis (H2) predicted that greater need frustration would show the complementary profile-specific pattern: greater likelihood of persistence in or movement toward controlled or amotivated configurations and lower likelihood of movement from low regulation toward profiles characterized by stronger identified or intrinsic regulation. These hypotheses concerned theoretically interpretable origin–destination contrasts rather than an assumption that the empirically derived profiles formed a single ordinal hierarchy. The third hypothesis (H3) predicted that, relative to stability in the same origin profile, movement toward a more autonomous configuration would be associated with greater subsequent leisure-time exercise, whereas movement toward a less internalized configuration would be associated with lower subsequent exercise. Together, the exploratory profile and transition description under RQ1 and the theory-driven hypotheses under RQ2 and RQ3 shift the focus from static exercise-motivation types to a dynamic need–transition–behavior process.

## 2 Methods

### 2.1 Study design and participants

This study used a three-wave prospective design to examine the configuration and short-term mobility of exercise motivation among Chinese college students. Data were collected at baseline (T1), approximately 28 days later (T2), and approximately 28 days after T2 (T3), yielding an observation period of approximately 8 weeks. Data collection was calendar-aligned across the participating colleges, with all participating classes completing T1 in October 2022, T2 in November 2022, and T3 in December 2022; participants therefore did not enter the study in staggered recruitment waves. T1 occurred during the early part of the academic term, T2 during the middle of the term, and T3 toward the end of the term. None of the scheduled assessments coincided with final examinations or major holiday periods. The same measures of exercise regulation, exercise-specific psychological need satisfaction and frustration, and leisure-time exercise were administered at all three waves. The 4-week interval between adjacent waves aligned with the reference period used for the psychological-need measure and was intended to capture short-term changes in motivational configurations rather than long-term developmental trajectories. Because the three assessments occupied fixed points within a single academic period, wave-to-wave changes should be interpreted in the context of that shared academic calendar.

Participants were recruited from six colleges in Jiangsu Province, China. The participating institutions were contacted through established professional relationships between members of the research team and college instructors and thus represented a convenience sample of colleges. Paper-and-pencil questionnaires were administered during required physical education classes at all three waves. Participation in the research was voluntary and was unrelated to course assessment. Before data collection, students were informed that they could decline to participate, omit any questionnaire item, or withdraw from the study at any time without penalty or other negative consequences. Ethical approval was obtained from the Institutional Review Board of Yancheng Teachers University, and informed consent was obtained from all participants before data collection. The sample size was determined pragmatically by the accessible cohort of students in the participating classes. A conventional *a priori* power calculation was not conducted because the number and separation of latent profiles were unknown before analysis, and simulation evidence indicates that profile-recovery power depends jointly on profile separation, the number of indicators, and sample size rather than on a single effect size (Tein et al., 2013). The achieved sample was therefore evaluated empirically using profile size, classification quality, random-start reproducibility, and posterior expected transition counts, with prespecified count thresholds applied to the follow-up transition analyses. At T1, the baseline analytical cohort comprised 1,780 unique students. Follow-up participation and patterns of missing data are presented in the Results section.

### 2.2 Measures

#### 2.2.1 Exercise regulation

Exercise regulation was assessed at T1, T2, and T3 using the 18-item Chinese-translated Behavioral Regulation in Exercise Questionnaire–2 (C-BREQ-2; Liu et al., 2015), which was derived from the BREQ-2 developed by Markland and Tobin (2004). The BREQ-2 assesses qualitatively distinct reasons for engaging, or not engaging, in exercise. Liu et al. reported support for an 18-item, five-factor C-BREQ-2 among university students from Mainland China and Hong Kong, including evidence concerning factorial structure, internal consistency, discriminant and nomological validity, and measurement invariance across the two samples. Integrated regulation was not assessed because it is not included in the BREQ-2/C-BREQ-2. This omission reflects the measurement history of the instrument rather than a data-driven exclusion in the present study or an assumption that integrated regulation is theoretically unimportant. During the early development of the BREQ, integrated regulation proved difficult to distinguish empirically from the adjacent identified and intrinsic forms of motivation and consequently was not included in the BREQ-2. Subsequent work developed a separate integrated-regulation subscale and provided support for its psychometric and theoretical validity; these items were subsequently incorporated into the BREQ-3 (Wilson et al., 2006; Zamarripa et al., 2018). The present data collection used the established 18-item C-BREQ-2 validated in Chinese university students; consequently, no integrated-regulation items were available for the present profile analyses. The five regulation dimensions were amotivation, external regulation, introjected regulation, identified regulation, and intrinsic regulation. Amotivation, external regulation, and intrinsic regulation were assessed with four items each; introjected and identified regulation were assessed with three items each. Participants were asked to indicate how true each statement was for their reasons for exercising “at the present time.” Responses ranged from 0 (not true for me) to 4 (very true for me). Illustrative items included “I exercise because other people say I should” for external regulation, “I feel guilty when I don’t exercise” for introjected regulation, “I value the benefits of exercise” for identified regulation, “I exercise because it’s fun” for intrinsic regulation, and “I don’t see the point in exercising” for amotivation.

#### 2.2.2 Exercise-specific psychological need satisfaction and frustration

Exercise-specific psychological need experiences were assessed at each wave using an exercise-context adaptation of the 24-item Basic Psychological Need Satisfaction and Frustration Scale (BPNSFS). The general BPNSFS was developed and evaluated across Belgium, China, the United States, and Peru. Its Chinese-language item pool was produced through translation and independent back-translation, and the final 24-item, six-factor model demonstrated measurement equivalence across countries (Chen et al., 2015). In the present study, the existing Chinese BPNSFS items were contextualized to participants’ experiences while exercising or participating in leisure-time physical activity during the preceding 4 weeks. Exercise-context research has supported a six-factor representation that separately assesses satisfaction and frustration of autonomy, competence, and relatedness (Rodrigues et al., 2019). Because the exact Chinese exercise-context adaptation had not undergone a separate validation before data collection, its internal consistency, wave-specific factor structure, and longitudinal measurement invariance were evaluated in the present sample.

The six four-item dimensions were autonomy satisfaction, autonomy frustration, competence satisfaction, competence frustration, relatedness satisfaction, and relatedness frustration. Example items included “When exercising, I feel a sense of choice and freedom in what I do” for autonomy satisfaction, “Most of my exercise participation feels like something I have to do” for autonomy frustration, “I feel confident that I can exercise well” for competence satisfaction, “I have serious doubts about whether I can exercise well” for competence frustration, “I feel connected with people who care for me during exercise” for relatedness satisfaction, and “I feel excluded from the exercise group I want to belong to” for relatedness frustration. Responses ranged from 1 (not true at all) to 5 (completely true).

#### 2.2.3 Leisure-time exercise

Leisure-time exercise was assessed at all three waves using a Chinese-language version of the Godin Leisure-Time Exercise Questionnaire (GLTEQ; Godin and Shephard, 1985). A Chinese translation of the same weekly leisure-time exercise format has previously been developed through bilingual expert review and pilot testing and administered among Chinese college students (Keating et al., 2006); the GLTEQ has also been used among university students from Mainland China and Hong Kong (Liu et al., 2015). Participants reported the number of times during a typical seven-day period that they engaged in strenuous, moderate, and mild or light exercise for more than 15 min during their free time. The questionnaire provided examples for each intensity category. The GLTEQ was designed as a brief self-report measure of typical leisure-time exercise, and the traditional leisure score weights strenuous, moderate, and light exercise frequencies by 9, 5, and 3, respectively (Godin, 2011). The weighted weekly score was calculated by summing nine times the strenuous-exercise frequency, five times the moderate-exercise frequency, and three times the light-exercise frequency. Higher scores indicated a greater weighted frequency of leisure-time exercise.

### 2.3 Statistical analysis

#### 2.3.1 Preliminary and measurement analyses

Participant characteristics, retention, missingness, and study-variable distributions were examined using descriptive statistics. Within-wave Pearson correlations were calculated among the five exercise-regulation dimensions, need satisfaction, need frustration, and GLTEQ. Internal consistency was evaluated at each wave using Cronbach’s alpha and model-based composite reliability. Potential attrition bias was examined by comparing participants with and without T3 data on baseline study variables and demographic characteristics using standardized mean or proportion differences; a supplementary logistic regression examined baseline predictors of T3 participation.

Before person-centered analyses, the measurement structures of the C-BREQ-2 and exercise-specific BPNSFS were evaluated at each wave using confirmatory factor analysis with the weighted least-squares mean- and variance-adjusted estimator and theta parameterization. The C-BREQ-2 specified five correlated regulation factors, whereas the BPNSFS specified six correlated need-satisfaction and need-frustration factors. A higher-order BPNSFS model was additionally tested to support the use of overall need satisfaction and need frustration in subsequent analyses. Model fit was evaluated using the comparative fit index (CFI), Tucker–Lewis index (TLI), root mean square error of approximation (RMSEA), and standardized root mean square residual (SRMR), together with the scaled chi-square statistic. Longitudinal measurement invariance was examined sequentially through configural, threshold, and threshold-plus-loading models, with same-item residual correlations permitted across waves. Model comparisons considered scaled chi-square difference tests and changes in practical fit indices (Chen, 2007; Wu and Estabrook, 2016).

#### 2.3.2 Profile enumeration and latent transition analysis

Supportive latent profile analyses were first conducted separately at T1, T2, and T3 using the five C-BREQ-2 subscale means as indicators. One- through six-profile solutions were examined under equal diagonal and equal unrestricted covariance structures. Solutions were compared using information criteria, entropy, profile size, classification quality, and substantive interpretability. These wave-specific analyses were used to determine whether broadly comparable configurations could be identified independently at each assessment occasion and therefore served as supportive checks. The longitudinal model remained the primary basis for selecting and interpreting the longitudinal profile structure. Consistent with RQ1, profile enumeration and the relative pattern of profile stability were interpreted as exploratory or descriptive features of the fitted models rather than as formal confirmatory hypothesis tests.

The primary person-centered analysis was a three-wave continuous-indicator latent transition analysis (Collins and Lanza, 2010). To ensure that transitions represented movement between profiles with the same substantive meaning at each assessment occasion, time-invariant profile definitions were deliberately imposed by constraining profile-specific indicator means and the common within-profile covariance structure to equality across T1, T2, and T3. Separate transition matrices were estimated for T1 → T2 and T2 → T3. One- through six-profile models were estimated by maximum likelihood using all available profile-indicator data under a missing-at-random assumption. Models with two through six profiles initially used one deterministic and 19 random starts; the leading five- and six-profile solutions were subsequently evaluated with 100 independent starts.

Among the one- through six-profile models evaluated, the retained solution was selected from the combined evidence of information criteria, entropy, smallest-profile size, replication of the best log-likelihood, substantive distinctiveness, and agreement with the wave-specific analyses rather than any single statistic (Nylund et al., 2007). Information criteria were treated as complexity-penalized comparative indices rather than as raw measures of fit, and no single numerical or visual criterion was treated as determinative. Scree plots of the BIC trajectories for the evaluated solutions are provided in Supplementary Figure S3 to complement the numerical information criteria and facilitate visual assessment of changes in relative model fit across profile counts. These plots were used as descriptive aids to model comparison rather than as a separate model-selection rule. The one- through six-profile range also encompassed the four- to six-profile structures reported in the most directly relevant prior exercise-motivation studies. Because six profiles constituted the upper boundary of the evaluated candidate set, the retained model is interpreted as the best-supported solution among those examined rather than as evidence that more complex solutions could not exist. For reproducible reporting, profiles were ordered using an internalization index calculated as the mean of identified and intrinsic regulation minus the mean of external regulation, introjected regulation, and amotivation. The index was used only to determine reporting order and was not entered into model estimation or profile selection. Posterior membership and joint origin–destination probabilities were retained for subsequent analyses. Profile prevalence and expected transition counts were derived from these posterior probabilities, whereas modal classifications were used only to describe classification quality.

#### 2.3.3 Missing data and psychological-need predictors of transitions

Missing profile indicators were handled directly within the maximum-likelihood latent transition model. Missing manifest predictors, covariates, and GLTEQ components used in follow-up analyses were addressed with multiple imputation by chained equations. Fifty imputed data sets were generated with 20 iterations each, using regulation scores, need satisfaction and frustration, GLTEQ variables across waves, demographic variables, and follow-up participation indicators. Predictive mean matching was used for continuous variables. Latent profile membership was not imputed, and results were pooled across imputations using Rubin’s rules (Rubin, 1987; van Buuren and Groothuis-Oudshoorn, 2011). After the final latent transition solution was fixed, psychological-need predictors were examined by comparing each eligible transition with stability in the same origin profile. Origin–destination contrasts were retained rather than aggregating destinations into “more” versus “less” autonomous categories because the latent profiles represented qualitatively different multivariate configurations and did not constitute a prespecified ordinal scale. In particular, profiles could simultaneously contain comparatively autonomous and controlled regulations. The internalization index used to order profiles for reporting was therefore not used to define an inferential hierarchy or to collapse transition outcomes. This approach preserved the theoretical meaning of the starting configuration and specific destination while avoiding a *post hoc* ordinal assumption about the latent states. Comparisons required a posterior expected destination count of at least 10 and an expected stable-profile count of at least 50. Posterior joint transition probabilities were used as fractional outcomes and weights rather than assigning individuals to error-free transition categories. Quasibinomial logistic models simultaneously included origin-wave need satisfaction and need frustration, with T1 predictors used for T1 → T2 transitions and T2 predictors for T2 → T3 transitions. Predictors were standardized using pooled T1 and T2 distributions, and HC3 robust covariance estimates were used. Sensitivity models additionally controlled origin-wave log-transformed GLTEQ. The resulting probabilities refer to the selected destination transition relative to same-origin stability and should not be interpreted as the unconditional transition probabilities from the latent transition model.

#### 2.3.4 Profile transitions and later exercise

Prospective behavioral analyses examined whether T1 → T2 profile transitions predicted T3 leisure-time exercise. T2 → T3 transitions were not used because their destination profile and the exercise outcome were both assessed at T3. Eligible transitions were compared with stability in the same origin profile using posterior joint transition probabilities as fractional weights. The primary weighted linear regression predicted log-transformed T3 GLTEQ while controlling log-transformed T1 GLTEQ. HC1 robust standard errors were clustered by participant to account for individuals contributing fractional information to both transition and stability possibilities. Adjusted T3 estimates were back-transformed for interpretation, and transition effects were expressed as ratios of GLTEQ + 1 for movers relative to same-origin stayers. Two sensitivity analyses were conducted: one additionally controlled T2 GLTEQ, and the other modeled raw T3 GLTEQ while controlling raw T1 GLTEQ. Estimates were pooled across the 50 imputed data sets using Rubin’s rules.

Analyses were conducted in R version 4.5.0 (R Core Team, 2025). Confirmatory factor analyses were estimated with *lavaan* (Rosseel, 2012), wave-specific latent profile analyses with *mclust* (Scrucca et al., 2016), longitudinal latent transition models with *LMest* (Pennoni et al., 2024), and multiple imputation with *mice* (van Buuren and Groothuis-Oudshoorn, 2011). HC3 heteroskedasticity-consistent covariance estimates were based on MacKinnon and White (1985), and participant-clustered covariance estimates were informed by Cameron and Miller (2015). All tests were two-sided and 95% confidence intervals were reported. Holm corrections controlled familywise error across the prespecified transition comparisons (Holm, 1979). For the psychological-need analyses, corrections were applied separately by transition interval, model specification, and predictor family; for the behavioral analyses, they were applied across eligible transitions within each outcome specification. Confirmatory interpretation considered Holm-adjusted *p* values below 0.05 together with effect magnitude, confidence intervals, expected transition-cell sizes, and consistency across sensitivity analyses. Posterior joint probabilities allowed the follow-up analyses to retain uncertainty in profile and transition classification. However, parameters of the selected latent transition model were treated as fixed and their estimation uncertainty was not propagated into the subsequent regression standard errors. Accordingly, the follow-up estimates were interpreted as posterior-probability-weighted pairwise associations rather than results from a fully integrated one-step conditional latent transition model. The study hypotheses and analysis plan were not preregistered, and no time-stamped public protocol was deposited before analysis. Preregistration was not incorporated into the original study workflow; accordingly, the exploratory status of profile enumeration under RQ1 and the non-preregistered status of the theory-guided hypotheses under RQ2 and RQ3 are stated explicitly and should be considered when interpreting the evidential strength of the findings.

## 3 Results

### 3.1 Participant flow, missing data, and descriptive statistics

The analytical sample comprised 1,780 students from six colleges. Participants had a mean age of 19.67 years (SD = 1.21, range = 18–23), and 52.9% were female. Students were distributed across all 4 years of study: 654 (36.7%) were in Year 1, 516 (29.0%) in Year 2, 433 (24.3%) in Year 3, and 177 (9.9%) in Year 4. Thus, the sample was not restricted to a single academic year. Retained data were available for 1,692 students (95.1%) at T2 and 1,575 (88.5%) at T3; 1,555 students (87.4%) contributed data at all three waves (Table 1). Missingness increased across waves but remained modest relative to follow-up retention. Multiple imputation produced 50 completed data sets for the manifest predictors, covariates, and GLTEQ components used in the follow-up analyses (Supplementary Table S1). Mean levels of the exercise-regulation and psychological-need variables were broadly similar across waves, as were mean GLTEQ scores (Table 1; Supplementary Table S2). Baseline differences between students with and without T3 data were generally small. The largest absolute standardized difference was 0.205 for identified regulation. In the supplementary retention model, T1 identified regulation was the only baseline variable significantly associated with T3 participation, OR = 1.25, 95% CI [1.02, 1.53], *p* = 0.032 (Supplementary Table S3).

**Table 1:** Participant characteristics, retention, and descriptive statistics for the primary study variables.

A. Participant characteristics and retention			
Characteristic	Value	Characteristic	Value
Total sample	1,780	Age, M (SD), range	19.67 (1.21), 18–23
Female	941 (52.9%)	Male	839 (47.1%)
Year 1	654 (36.7%)	Year 2	516 (29.0%)
Year 3	433 (24.3%)	Year 4	177 (9.9%)
Institution 1	330 (18.5%)	Institution 2	310 (17.4%)
Institution 3	285 (16.0%)	Institution 4	290 (16.3%)
Institution 5	280 (15.7%)	Institution 6	285 (16.0%)
T2 retained-data present	1,692 (95.1%)	T3 retained-data present	1,575 (88.5%)
All three waves present	1,555 (87.4%)	Intermittent T1 + T3 (no T2)	20 (1.1%)

Panel B. Primary study variables by wave									
Construct	T1			T2			T3		
	*n*	M (SD)	*α*	*n*	M (SD)	*α*	*n*	M (SD)	*α*
Amotivation	1,777	1.45 (1.01)	0.87	1,687	1.44 (1.03)	0.88	1,570	1.50 (1.02)	0.87
External regulation	1,776	1.40 (0.91)	0.81	1,690	1.46 (0.95)	0.83	1,569	1.50 (0.92)	0.80
Introjected regulation	1,777	2.52 (0.87)	0.77	1,688	2.54 (0.91)	0.83	1,575	2.48 (0.86)	0.76
Identified regulation	1,777	2.68 (0.92)	0.75	1,692	2.60 (0.89)	0.68	1,575	2.62 (0.93)	0.74
Intrinsic regulation	1,773	2.65 (0.98)	0.84	1,690	2.60 (0.98)	0.85	1,574	2.64 (0.96)	0.85
Need satisfaction	1,773	3.56 (0.61)	0.86	1,682	3.56 (0.62)	0.86	1,569	3.54 (0.61)	0.86
Need frustration	1,774	2.48 (0.69)	0.89	1,678	2.54 (0.68)	0.88	1,570	2.48 (0.69)	0.90
Weighted GLTEQ	1,742	41.14 (26.64)	—	1,660	41.03 (33.79)	—	1,547	42.51 (38.41)	—

Values in Panel A are *n* (%) unless otherwise indicated. M, mean; SD, standard deviation; *α*, Cronbach’s alpha; GLTEQ, Godin Leisure-Time Exercise Questionnaire weighted score. *α* was not calculated for GLTEQ because it is a behavioral index rather than a reflective scale. GLTEQ medians [interquartile ranges] were 36 [22, 55] at T1, 32 [17, 54] at T2, and 32 [18, 53] at T3. The observed *n* varies because of item-level and wave-level missingness.

### 3.2 Measurement quality and longitudinal comparability

Reliability estimates were generally acceptable across constructs and waves, with Cronbach’s alpha ranging from 0.68 to 0.90 and model-based composite reliability from 0.68 to 0.89 (Supplementary Table S4). The hypothesized five-factor C-BREQ-2 and six-factor BPNSFS models showed good fit at each wave, and the higher-order BPNSFS model also showed acceptable fit, supporting the use of overall need satisfaction and need frustration in the transition analyses (Table 2, Panel A). Longitudinal invariance analyses further supported comparability across T1, T2, and T3. Threshold and loading constraints were supported for the C-BREQ-2. For the BPNSFS, the threshold-plus-loading comparison produced a significant scaled chi-square difference, but changes in practical fit were negligible (ΔCFI = −0.00031; ΔRMSEA = 0.00017; Table 2, Panel B). Complete measurement results are reported in Supplementary Tables S4, S5. Together, these findings provided study-specific support for the internal structure and longitudinal comparability of the Chinese exercise-context BPNSFS adaptation.

**Table 2:** Wave-specific measurement-model fit and longitudinal measurement invariance.

Panel A. Wave-specific confirmatory factor analyses							
Instrument and model	Wave	*n*	Scaled chi-square (df)	CFI	TLI	RMSEA [90% CI]	SRMR
C-BREQ-2, five correlated factors	T1	1,780	367.5 (125)	0.991	0.989	0.033 [0.029, 0.037]	0.027
C-BREQ-2, five correlated factors	T2	1,692	321.3 (125)	0.993	0.992	0.030 [0.026, 0.035]	0.025
C-BREQ-2, five correlated factors	T3	1,575	330.0 (125)	0.991	0.989	0.032 [0.028, 0.037]	0.027
BPNSFS, six correlated factors	T1	1,780	455.8 (237)	0.993	0.992	0.023 [0.020, 0.026]	0.024
BPNSFS, six correlated factors	T2	1,692	572.3 (237)	0.989	0.987	0.029 [0.026, 0.032]	0.028
BPNSFS, six correlated factors	T3	1,575	437.7 (237)	0.994	0.992	0.023 [0.020, 0.027]	0.024
BPNSFS, six first-order + two higher-order factors	T1	1,780	592.8 (245)	0.989	0.988	0.028 [0.025, 0.031]	0.028
BPNSFS, six first-order + two higher-order factors	T2	1,692	742.9 (245)	0.983	0.981	0.035 [0.032, 0.038]	0.033
BPNSFS, six first-order + two higher-order factors	T3	1,575	584.4 (245)	0.989	0.988	0.030 [0.027, 0.033]	0.030

Panel B. Longitudinal measurement-invariance tests										
Instrument	Model	Scaled chi-square (df)	CFI	TLI	RMSEA	SRMR	Delta CFI	Delta RMSEA	Delta chi-square (df)	*p*
C-BREQ-2	Configural	1800.6 (1218)	0.992	0.991	0.016	0.023	—	—	—	—
C-BREQ-2	Thresholds	1876.3 (1290)	0.992	0.991	0.016	0.023	−0.00005	−0.00041	69.5 (72)	0.563
C-BREQ-2	Thresholds + loadings	1886.6 (1316)	0.992	0.992	0.016	0.023	0.00021	−0.00037	34.9 (26)	0.115
BPNSFS	Configural	2840.7 (2259)	0.993	0.992	0.012	0.023	—	—	—	—
BPNSFS	Thresholds	2942.2 (2355)	0.993	0.992	0.012	0.023	−0.00006	−0.00019	105.2 (96)	0.245
BPNSFS	Thresholds + loadings	3004.1 (2391)	0.993	0.992	0.012	0.023	−0.00031	0.00017	65.9 (36)	0.002

C-BREQ-2, Chinese Behavioral Regulation in Exercise Questionnaire-2; BPNSFS, Basic Psychological Need Satisfaction and Frustration Scale; CFI, comparative fit index; TLI, Tucker-Lewis index; RMSEA, root mean square error of approximation; SRMR, standardized root mean square residual. Item-level models used the WLSMV estimator with ordered indicators. Delta values compare each model with the immediately less constrained model. The BPNSFS threshold-to-loading scaled chi-square difference was statistically significant, but the changes in CFI and RMSEA were negligible.

### 3.3 Exercise-regulation profiles

Among the one- through six-profile solutions examined at each wave, supportive wave-specific latent profile analyses showed some variation across covariance specifications rather than identifying a single unequivocal stopping point. BIC was minimized at six profiles in five of the six wave-by-covariance combinations and at four profiles in the remaining combination (Supplementary Table S6; Supplementary Figure S3). Similarly, among the one- through six-profile longitudinal models evaluated, the six-profile solution provided the most favorable information criteria and remained substantively interpretable, with adequate profile size and classification quality. Although the largest BIC reduction occurred from one to two profiles, the longitudinal BIC trajectory did not show a unique elbow at two profiles: BIC decreased by 552.34 points from two to three profiles, by 756.14 points from three to four profiles, by 135.78 points from four to five profiles, and by a further 48.06 points from five to six profiles. Thus, substantial improvement remained beyond the two-profile solution, particularly from three to four profiles (Table 3; Supplementary Figure S3). The six-profile model had BIC = 58,442.25, entropy of 0.801, and a smallest profile representing approximately 6% of the sample. Focused random-start analyses also supported recovery of the retained solution (Table 3; Supplementary Table S7). Selection of six profiles therefore reflected the convergence of information criteria, classification quality, profile size, solution reproducibility, and substantive differentiation rather than the minimum BIC alone. The six profiles were labeled P1 amotivated–controlled, P2 low-regulation, P3 mixed intrinsic–controlled, P4 identified-dominant, P5 intrinsic-dominant, and P6 high autonomous–introjected (Figure 1A). P1 combined high amotivation with elevated external and introjected regulation; P2 showed generally low levels across regulations; P3 combined high intrinsic and elevated introjected regulation with moderate controlled regulation and amotivation; P4 was distinguished by high identified regulation; P5 by high intrinsic regulation together with low amotivation and external regulation; and P6 by very high identified and intrinsic regulation, high introjected regulation, and low amotivation and external regulation. Complete profile means and classification indices are provided in Supplementary Table S8.

**Table 3:** Enumeration and confirmation of the longitudinal exercise-regulation profile solution.

*K*	Log-likelihood	Parameters	AIC	BIC	Adjusted BIC	Entropy	Smallest state	Successful starts	LL starts within 0.01
1	−31084.46	20	62208.91	62318.60	62255.06	1.000	100.0%	1/1	1
2	−29855.02	30	59770.04	59934.57	59839.27	0.850	40.6%	20/20	20
3	−29526.46	44	59140.92	59382.23	59242.45	0.828	13.6%	20/20	19
4	−29081.03	62	58286.06	58626.09	58429.12	0.832	11.7%	20/20	20
5	−28930.81	84	58029.63	58490.31	58223.45	0.835	5.6%	100/100	58
6	−28809.49	110	57838.97	58442.25	58092.79	0.801	6.0%	100/100	21

*K*, number of latent profiles; AIC, Akaike information criterion; BIC, Bayesian information criterion; adjusted BIC, sample-size-adjusted BIC; LL, log-likelihood. Lower information criteria indicate better relative fit. The five- and six-profile solutions were subjected to a focused confirmation using 100 independent starts; models with one to four profiles used the original start set. The selected six-profile solution is shown in bold. In the focused confirmation, the best six-profile log-likelihood was recovered within 0.01 units in 21 starts and within 0.05 units in 29 starts.

**Figure 1:**
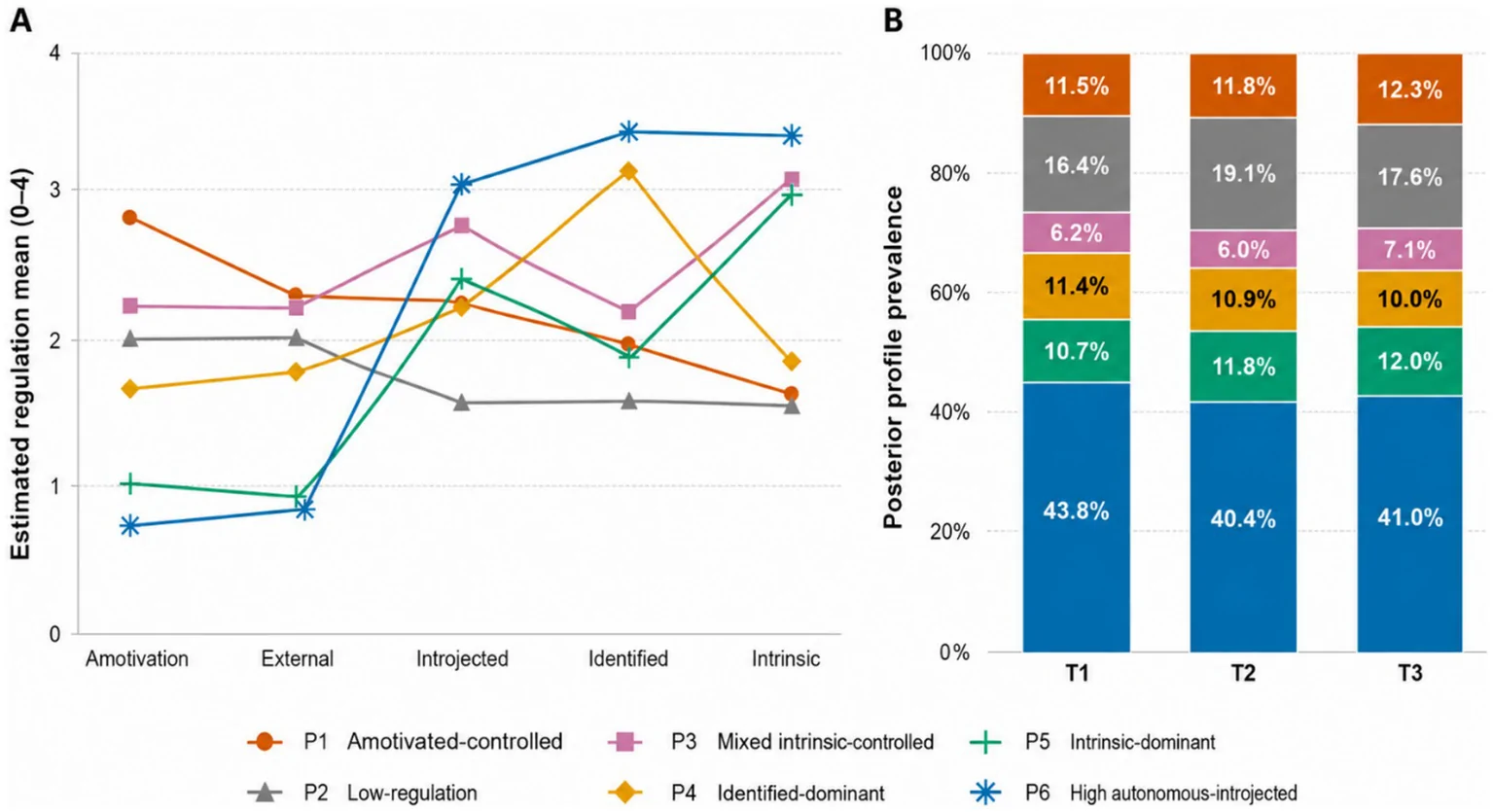
Final six-profile exercise-regulation solution and posterior profile prevalence across waves. Panel **(A)** shows the model-estimated means for amotivation, external regulation, introjected regulation, identified regulation, and intrinsic regulation in the final six-profile latent transition solution. Panel **(B)** shows posterior profile prevalence at T1, T2, and T3. Profile means were constrained to have the same measurement definition across waves, whereas posterior prevalence was allowed to vary. P1 = amotivated–controlled; P2 = low-regulation; P3 = mixed intrinsic–controlled; P4 = identified-dominant; P5 = intrinsic-dominant; P6 = high autonomous–introjected.

### 3.4 Profile prevalence and transitions

Profile prevalence changed only modestly across the three waves (Figure 1B). P6 was the largest profile at every occasion, accounting for 43.8% of the posterior distribution at T1, 40.4% at T2, and 41.0% at T3, whereas P3 was consistently the smallest. The transition matrices indicated substantial, but not complete, short-term stability (Figure 2). Overall, 76.9% of posterior transition mass remained on the diagonal from T1 to T2 and 73.8% from T2 to T3. P6 was highly stable across both intervals, with stability probabilities of 79.1 and 80.5%, although P1 was the most stable profile during T1 → T2 (85.0%). P4 showed the lowest stability during T2 → T3 (55.6%). The most prominent off-diagonal movement was P2 → P6, occurring with probabilities of 18.0% from T1 to T2 and 20.1% from T2 to T3. Other notable transitions included P4 → P2, P5 → P6, and P6 → P2; during T2 → T3, P3 → P1 and P4 → P6 were also relatively common. Full transition matrices and posterior expected counts are reported in Supplementary Table S8.

**Figure 2:**
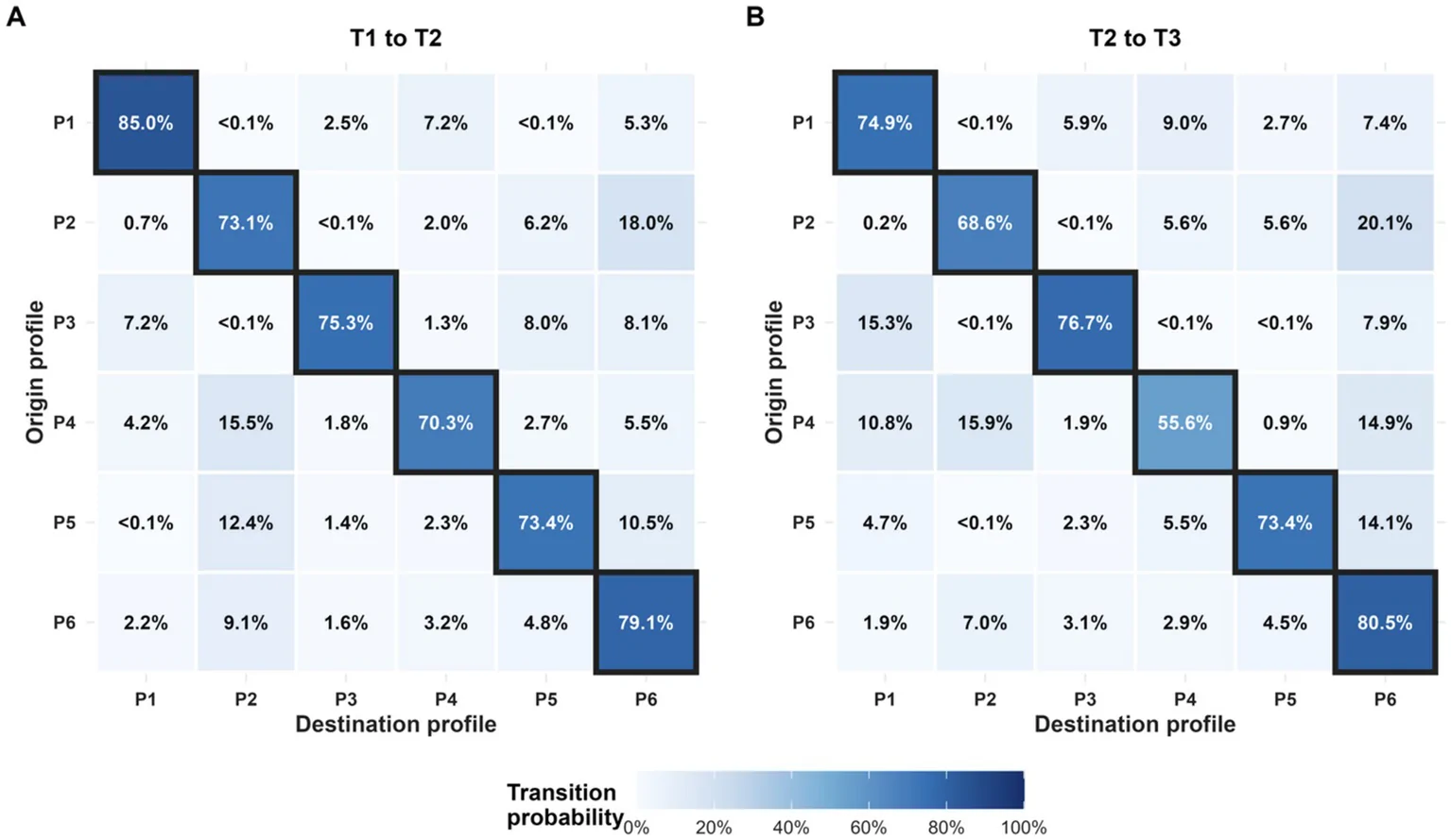
Model-estimated exercise-regulation profile transition probabilities across the two approximately 4-week intervals. Heatmaps show the model-estimated probabilities of destination-profile membership conditional on origin-profile membership for T1 to T2 in Panel **(A)** and T2 to T3 in Panel **(B)**. Rows represent origin profiles and columns represent destination profiles. Diagonal cells, outlined in black, represent profile stability. The values were obtained from the final six-profile latent transition model rather than from cross-tabulations of modal profile assignments. P1 = amotivated–controlled; P2 = low-regulation; P3 = mixed intrinsic–controlled; P4 = identified-dominant; P5 = intrinsic-dominant; P6 = high autonomous–introjected.

### 3.5 Psychological-need predictors of profile transitions

Thirty move-versus-stability comparisons met the prespecified expected-count requirements. Figure 3 presents the odds ratios and 95% confidence intervals for need satisfaction and need frustration across all eligible primary transition comparisons, including effects that did not remain statistically significant after multiplicity correction; Table 4 provides a concise summary of the Holm-significant effects. Four psychological-need effects remained significant after Holm correction and are highlighted in Figure 3. From T1 to T2, greater need frustration was associated with lower odds of moving from P2 low-regulation to P5 intrinsic-dominant rather than remaining in P2, OR = 0.41, 95% CI [0.27, 0.62], Holm-adjusted *p* < 0.001. Greater need satisfaction was associated with lower odds of moving from P5 intrinsic-dominant to P2, OR = 0.47, 95% CI [0.30, 0.76], Holm-adjusted *p* = 0.020, and from P6 high autonomous–introjected to P2, OR = 0.49, 95% CI [0.36, 0.67], Holm-adjusted *p* < 0.001. From T2 to T3, greater need frustration was associated with lower odds of moving from P2 low-regulation to P4 identified-dominant rather than remaining in P2, OR = 0.62, 95% CI [0.46, 0.84], Holm-adjusted *p* = 0.040. These four associations remained significant and were essentially unchanged after origin-wave GLTEQ was controlled. No other need-satisfaction or need-frustration effect survived multiplicity correction. Complete numerical estimates for the primary and prior-GLTEQ-adjusted models are reported in Supplementary Table S9, and Supplementary Figure S1 displays the corresponding sensitivity comparisons.

**Figure 3:**
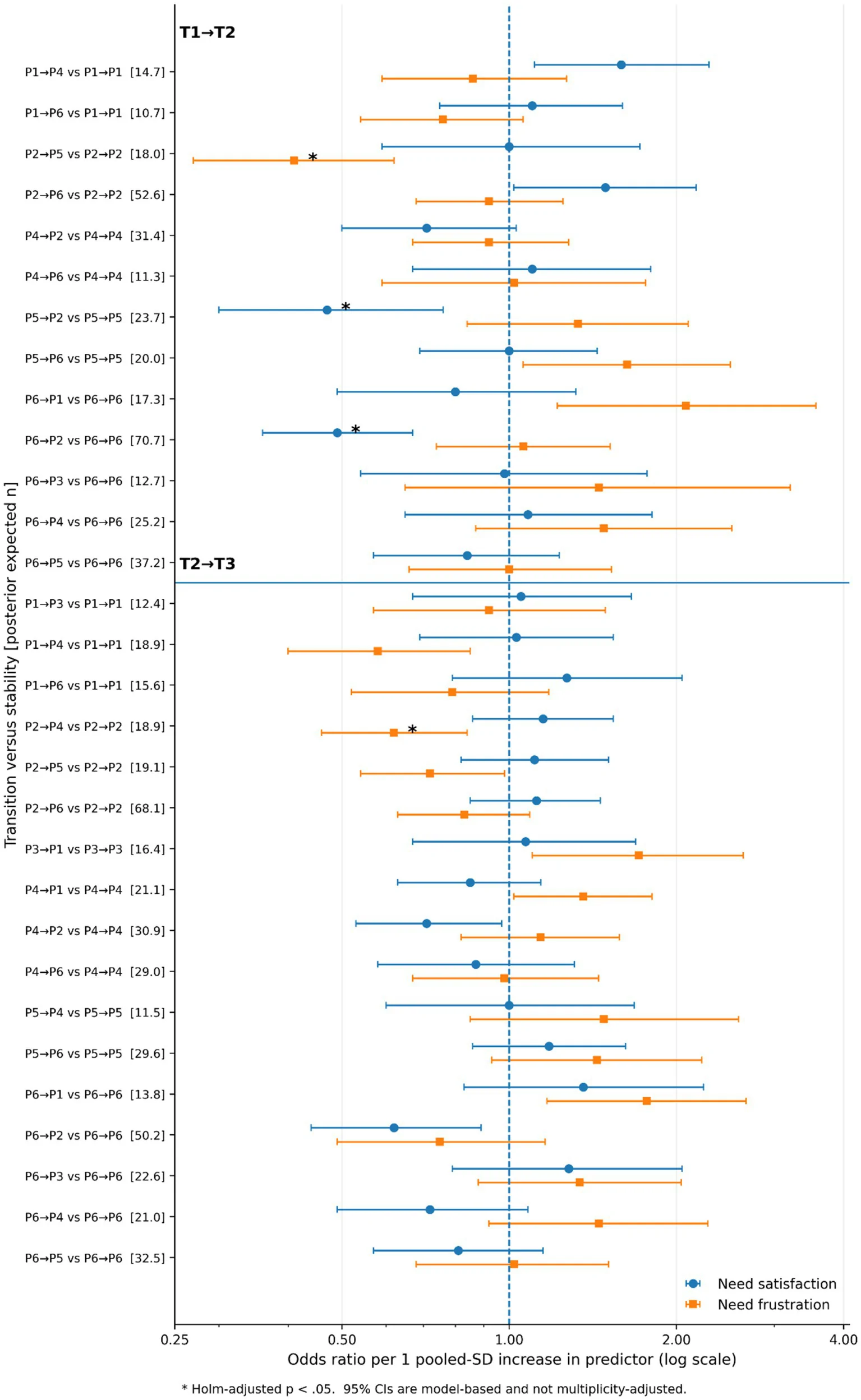
Associations of exercise-specific need satisfaction and need frustration with eligible profile transitions. Error-bar plots show odds ratios (ORs) and 95% confidence intervals (CIs) for all eligible primary move-versus-stability comparisons, including significant and nonsignificant effects. T1 → T2 comparisons appear in the upper portion, and T2 → T3 comparisons appear in the lower portion. All estimates use a common logarithmic odds-ratio scale; the vertical reference line at OR = 1.00 indicates no association. Blue circles represent need satisfaction and orange squares represent need frustration. Odds ratios represent a one-pooled-standard-deviation increase in the focal predictor, with need satisfaction and need frustration entered simultaneously. The plotted 95% CIs are model-based and are not adjusted for multiple comparisons; asterisks (*) identify effects with Holm-adjusted *p* < 0.05. Posterior expected transition counts are shown in brackets and may be fractional. P1 = amotivated–controlled; P2 = low-regulation; P3 = mixed intrinsic–controlled; P4 = identified-dominant; P5 = intrinsic-dominant; P6 = high autonomous–introjected.

**Table 4:** Holm-significant associations of exercise-specific need satisfaction and frustration with profile transitions.

Interval	Transition comparison	Expected n	Predictor	Primary OR [95% CI]	Holm *p*	Prior-GLTEQ-adjusted OR [95% CI]	Holm *p*
T1 → T2	P2 → P5 vs. P2 → P2	18.0	Need frustration	0.41 [0.27, 0.62]	<0.001	0.41 [0.27, 0.64]	<0.001
T1 → T2	P5 → P2 vs. P5 → P5	23.7	Need satisfaction	0.47 [0.30, 0.76]	0.020	0.47 [0.30, 0.76]	0.021
T1 → T2	P6 → P2 vs. P6 → P6	70.7	Need satisfaction	0.49 [0.36, 0.67]	<0.001	0.49 [0.36, 0.67]	<0.001
T2 → T3	P2 → P4 vs. P2 → P2	18.9	Need frustration	0.62 [0.46, 0.84]	0.040	0.61 [0.45, 0.84]	0.035

Only effects with Holm-adjusted *p* < 0.05 in the primary model are shown. Each odds ratio compares the selected destination transition with stability in the same origin profile and represents a one-standard-deviation increase in the predictor. The sensitivity model additionally controlled origin-wave log(1 + GLTEQ). Expected *n* values are posterior expected transition counts and can therefore be fractional. P1 = amotivated-controlled; P2 = low-regulation; P3 = mixed intrinsic-controlled; P4 = identified-dominant; P5 = intrinsic-dominant; P6 = high autonomous-introjected; GLTEQ = Godin Leisure-Time Exercise Questionnaire.

### 3.6 Early profile transitions and T3 leisure-time exercise

Thirteen T1 → T2 transition comparisons met the prespecified expected-count criteria. Seven contrasts were significant after Holm correction in the primary model controlling T1 GLTEQ (Table 5 and Figure 4). Movement into P6 from the two least-internalized profiles predicted greater subsequent exercise. Relative to remaining in the same origin profile, P1 → P6 was associated with higher T3 GLTEQ, ratio = 1.72, 95% CI [1.31, 2.25], Holm-adjusted *p* < 0.001, although this transition had a relatively small posterior expected count of 10.7. The strongest upward-transition result was P2 → P6, ratio = 1.81, 95% CI [1.47, 2.22], Holm-adjusted *p* < 0.001. Transitions toward less favorable configurations generally predicted lower subsequent exercise. P5 → P2 was associated with lower T3 GLTEQ, ratio = 0.69, 95% CI [0.54, 0.88], Holm-adjusted *p* = 0.021. Relative to remaining in P6, transitions from P6 to P1, P2, P3, and P4 were also associated with lower T3 GLTEQ, with ratios ranging from 0.52 to 0.63 and all Holm-adjusted *p*-values below 0.05. In contrast, P6 → P5 did not differ significantly from P6 stability, suggesting that not every movement away from P6 was associated with reduced exercise. P2 → P5, P4 → P2, P4 → P6, and P5 → P6 also did not remain significant after multiplicity correction.

**Table 5:** Prospective associations of T1 → T2 profile transitions with T3 leisure-time exercise.

Transition comparison	Expected *n*	Adjusted T3 GLTEQ: stay [95% CI]	Adjusted T3 GLTEQ: move [95% CI]	Primary ratio [95% CI]	Holm *p*	T1- and T2-adjusted ratio [95% CI]	Holm *p*
P1 → P4 vs. P1 → P1	14.7	20.8 [18.6, 23.3]	23.6 [19.2, 29.0]	1.13 [0.91, 1.39]	0.545	1.07 [0.89, 1.28]	0.971
P1 → P6 vs. P1 → P1	10.7	20.9 [18.7, 23.4]	36.6 [28.3, 47.2]	**1.72 [1.31, 2.25]**	**<0.001**	1.33 [1.03, 1.73]	0.309
P2 → P5 vs. P2 → P2	18.0	19.1 [17.0, 21.5]	25.0 [18.6, 33.5]	1.29 [0.96, 1.74]	0.277	1.17 [0.87, 1.57]	0.918
P2 → P6 vs. P2 → P2	52.6	19.1 [17.0, 21.5]	35.3 [29.4, 42.4]	**1.81 [1.47, 2.22]**	**<0.001**	**1.48 [1.22, 1.80]**	**<0.001**
P4 → P2 vs. P4 → P4	31.4	26.1 [23.0, 29.6]	19.4 [15.8, 23.9]	0.75 [0.61, 0.93]	0.055	0.86 [0.71, 1.03]	0.650
P4 → P6 vs. P4 → P4	11.3	26.2 [23.1, 29.7]	35.5 [28.9, 43.5]	1.34 [1.07, 1.68]	0.055	1.18 [0.95, 1.45]	0.650
P5 → P2 vs. P5 → P5	23.7	33.3 [29.8, 37.3]	22.7 [17.9, 28.9]	**0.69 [0.54, 0.88]**	**0.021**	0.81 [0.64, 1.03]	0.569
P5 → P6 vs. P5 → P5	20.0	33.6 [30.0, 37.5]	42.2 [35.0, 50.9]	1.25 [1.02, 1.53]	0.128	1.16 [0.95, 1.41]	0.650
P6 → P1 vs. P6 → P6	17.3	41.3 [38.7, 43.9]	20.8 [16.5, 26.2]	**0.52 [0.41, 0.65]**	**<0.001**	0.78 [0.63, 0.97]	0.260
P6 → P2 vs. P6 → P6	70.7	41.2 [38.7, 43.9]	22.7 [19.0, 27.2]	**0.56 [0.47, 0.67]**	**<0.001**	0.86 [0.74, 1.01]	0.500
P6 → P3 vs. P6 → P6	12.7	41.2 [38.7, 43.9]	25.5 [18.4, 35.3]	**0.63 [0.46, 0.86]**	**0.028**	0.71 [0.53, 0.95]	0.244
P6 → P4 vs. P6 → P6	25.2	41.2 [38.7, 43.8]	24.7 [18.5, 32.9]	**0.61 [0.46, 0.81]**	**0.005**	0.74 [0.57, 0.98]	0.309
P6 → P5 vs. P6 → P6	37.2	41.2 [38.7, 43.9]	37.9 [32.6, 44.0]	0.92 [0.79, 1.07]	0.545	1.00 [0.87, 1.16]	0.983

The primary model predicted log(1 + T3 GLTEQ) from the T1 → T2 move-versus-stay contrast while controlling log(1 + T1 GLTEQ). Adjusted T3 GLTEQ values are back-transformed model estimates. The ratio is the mover-to-stayer ratio for GLTEQ + 1. The stricter sensitivity model additionally controlled log(1 + T2 GLTEQ). Boldface indicates Holm-adjusted *p* < 0.05. Expected *n* values are posterior expected transition counts and can therefore be fractional. P1 = amotivated-controlled; P2 = low-regulation; P3 = mixed intrinsic-controlled; P4 = identified-dominant; P5 = intrinsic-dominant; P6 = high autonomous-introjected; GLTEQ = Godin Leisure-Time Exercise Questionnaire.

**Figure 4:**
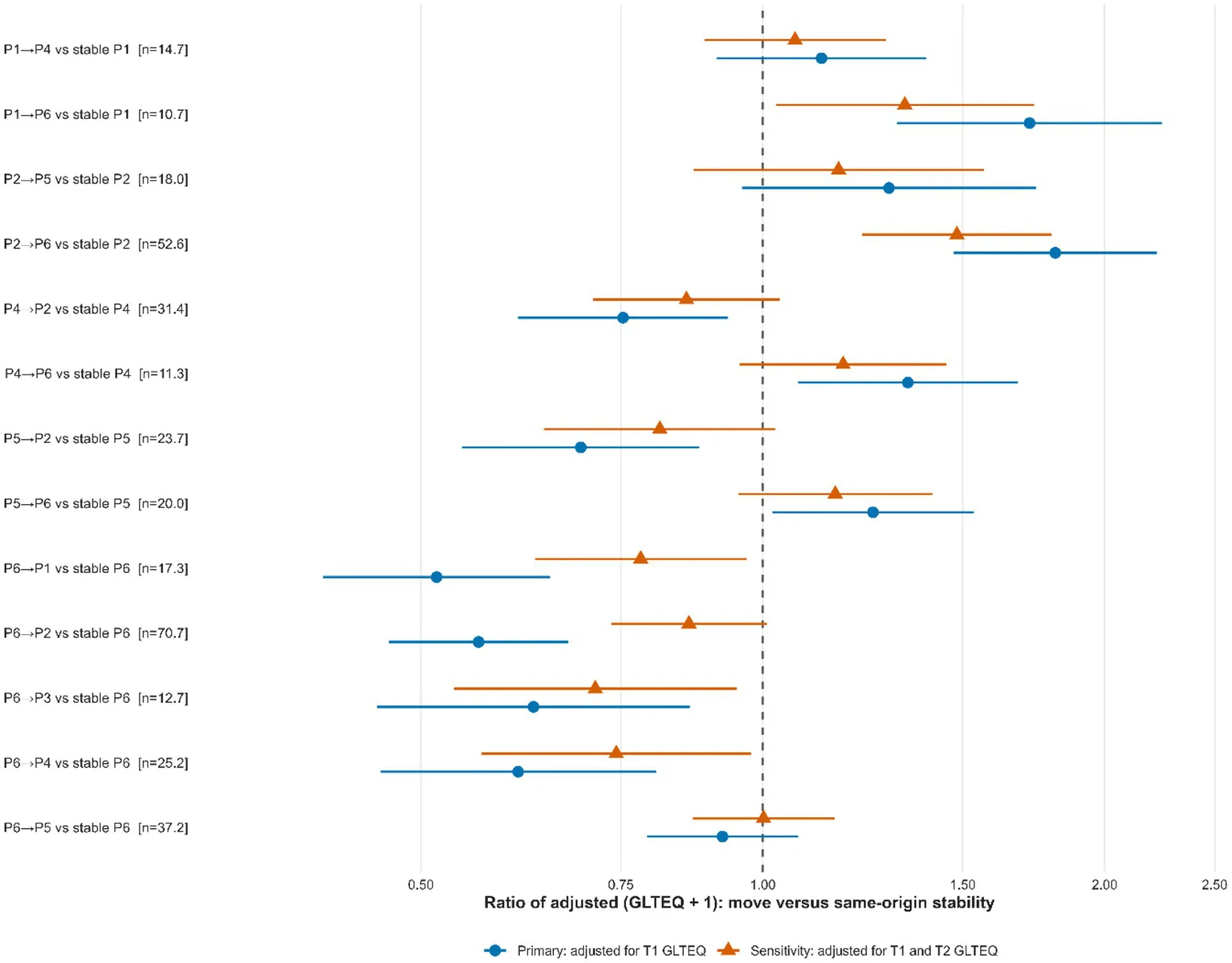
Prospective associations between T1-to-T2 exercise-regulation profile transitions and T3 leisure-time exercise. Points and 95% confidence intervals represent the adjusted ratio of T3 GLTEQ + 1 for students probabilistically represented by each T1-to-T2 transition relative to students remaining in the same origin profile. The primary model controlled T1 GLTEQ. The stricter sensitivity model additionally controlled T2 GLTEQ. Values greater than 1 indicate higher T3 leisure-time exercise among movers, whereas values below 1 indicate lower T3 exercise. Posterior expected transition counts are shown in brackets and can be fractional. P1 = amotivated–controlled; P2 = low-regulation; P3 = mixed intrinsic–controlled; P4 = identified-dominant; P5 = intrinsic-dominant; P6 = high autonomous–introjected.

The raw-GLTEQ sensitivity analysis reproduced the same seven Holm-significant contrasts (Supplementary Table S10; Supplementary Figure S2). When T2 GLTEQ was additionally controlled, however, effects were attenuated and only P2 → P6 remained significant, ratio = 1.48, 95% CI [1.22, 1.80], Holm-adjusted *p* < 0.001. Thus, the prospective associations were strongest for movement from low regulation into the high autonomous–introjected profile, whereas most other transition effects were reduced after exercise measured at the end of the transition interval was taken into account.

## 4 Discussion

This three-wave study examined the short-term configuration and mobility of exercise motivation among Chinese college students, the prospective roles of exercise-specific psychological-need experiences in profile transitions, and the associations of early transitions with later leisure-time exercise. Six profiles captured comparatively internalized, weakly internalized, and mixed combinations of regulation. Their population prevalence changed little across the 8-week study, yet meaningful individual movement occurred beneath this aggregate stability. Need satisfaction was most clearly associated with protection against movement from comparatively internalized profiles to low regulation, whereas need frustration was associated with a lower likelihood of movement from low regulation toward identified- or intrinsic-dominant profiles. Several early transitions predicted later exercise after baseline exercise was controlled, but movement from low regulation to the high autonomous–introjected profile was the only association that remained significant after exercise at the transition endpoint was additionally considered. Accordingly, exploratory RQ1 identified a six-profile longitudinal structure characterized by substantial but incomplete stability, whereas the three theory-driven hypotheses concerning psychological needs and subsequent exercise received partial and transition-specific support.

### 4.1 Exercise-regulation profiles and short-term mobility

The six-profile solution supports a configurational view of exercise motivation. The amotivated–controlled and low-regulation profiles represented distinct forms of weak internalization; the identified-dominant and intrinsic-dominant profiles were differentiated by personal value and enjoyment; and the mixed intrinsic–controlled and high autonomous–introjected profiles showed that autonomous and controlled regulations can coexist. This differentiation was not simply additional numerical subdivision. In particular, distinguishing the amotivated–controlled profile from the low-regulation profile separated students characterized by comparatively elevated amotivation and external/introjected regulation from those reporting generally low levels across all regulations. Likewise, the identified-dominant, intrinsic-dominant, mixed intrinsic–controlled, and high autonomous–introjected configurations reflected qualitatively different combinations of the five regulation dimensions rather than successive cuts on a single severity continuum. This broad pattern is consistent with previous person-centered studies identifying weakly motivated, autonomous, controlled, and mixed configurations in exercise populations, including Chinese college students (Emm-Collison et al., 2020; Gao et al., 2025; Lindwall et al., 2017; Zheng et al., 2026). Because the number and form of intermediate profiles vary across samples, however, the present solution should be viewed as the best-supported representation among the candidate models evaluated for this cohort, not as a uniquely correct or universal taxonomy.

The high autonomous–introjected profile was the largest profile at all three waves and was central to the behavioral findings. It combined very high identified and intrinsic regulation with high introjected regulation, but low external regulation and amotivation. Similar profiles have been reported in both Western and Chinese samples, reinforcing that strong autonomous motivation does not necessarily displace internal pressure (Gao et al., 2025; Lindwall et al., 2017; Zheng et al., 2026). Nevertheless, this profile should not be described as purely autonomous or uniformly optimal. The present analyses evaluated the complete configuration and cannot isolate whether introjected regulation contributed positively, negatively, or negligibly after the autonomous components were considered. Other person-centered evidence suggests that coexisting controlled motivation may accompany relatively high activity while weakening some wellbeing benefits (Claes et al., 2026). The comparatively favorable exercise pattern of this profile is therefore most defensibly attributed to its overall configuration, especially its very high identified and intrinsic regulation and low amotivation and external control.

The transition results further demonstrate why aggregate prevalence and individual mobility should be distinguished. Approximately three quarters of posterior transition probability remained within the same profile during each interval, but about one quarter reflected movement. The high autonomous–introjected profile was highly stable, consistent with evidence that strongly autonomous profiles can be comparatively enduring (Emm-Collison et al., 2020). However, the amotivated–controlled profile was most stable from T1 to T2, showing that stability is not synonymous with adaptiveness. The identified-dominant profile was least stable from T2 to T3, although the study did not measure the situational experiences that might explain this interval-specific pattern. Most notably, the largest off-diagonal movement in both intervals was directly from low regulation to high autonomous–introjected motivation. Organismic Integration Theory differentiates regulations by their degree of internalization, but empirically derived profiles are not fixed developmental stages that must be traversed sequentially (Deci and Ryan, 2000; Ryan and Deci, 2017). Taken together, these findings indicate that short-term stability was substantial but varied across profiles and intervals, while meaningful movement could occur directly between markedly different motivational configurations. The prominent low-regulation-to-high autonomous–introjected transition therefore illustrates mobility within the profile system without implying that the profiles constitute sequential developmental stages or that transition probabilities represent motivational distance.

### 4.2 Psychological needs as transition-specific antecedents

Need satisfaction appeared to operate primarily as a protective resource. Higher satisfaction was associated with lower odds of moving from the intrinsic-dominant or high autonomous–introjected profile to low regulation. Experiences of choice, effectiveness, and connection may therefore help preserve personally valued or enjoyable reasons for exercise and reduce motivational deterioration. This interpretation accords with Basic Psychological Needs Theory and prior exercise research linking need satisfaction with more self-endorsed regulation (Ryan and Deci, 2017; Vansteenkiste et al., 2020; Weman-Josefsson et al., 2015). The need-satisfaction hypothesis was nevertheless only partially supported because satisfaction did not consistently predict all favorable transitions. Its clearest role was the maintenance of internalized motivation already present rather than the initiation of every upward movement.

Need frustration showed a complementary but not simply opposite pattern. Greater frustration was associated with lower odds of moving from low regulation to intrinsic-dominant or identified-dominant motivation. Active experiences of coercion, inadequacy, or exclusion may therefore function as an internalization barrier, preventing students with weak motivation from developing more personally meaningful or enjoyable reasons for exercise. This pattern supports distinguishing need frustration from merely low need satisfaction and is consistent with evidence that the two experiences can coexist and relate differently to exercise regulation (Chen et al., 2015; Rodrigues et al., 2021; Vansteenkiste et al., 2020).

The four significant need effects remained essentially unchanged after prior exercise was controlled. However, only four of 30 eligible comparisons survived multiplicity correction, and the frequent, behaviorally robust transition from low regulation to high autonomous–introjected motivation was not predicted by the broad need composites. Psychological needs therefore explained selected transitions rather than motivational mobility as a whole. This transition specificity is substantively important because movements between multidimensional profiles need not represent interchangeable increments or decrements in autonomy. The findings therefore support an origin-dependent account in which the relevance of need experiences can differ according to both the motivational configuration from which students begin and the configuration toward which they move. At the same time, the present analyses should not be interpreted as estimating a single omnibus effect of psychological needs on all “upward” or all “downward” motivational movement. Changes in enjoyment, perceived value, social climate, exercise opportunities, or life circumstances may also be involved and should be measured directly in future research. The present findings identify connected prospective associations, not a mediated causal pathway.

### 4.3 Motivational transitions and later exercise

Early motivational movement was prospectively associated with later leisure-time exercise, but the pattern was origin- and destination-specific. Movement from the amotivated–controlled or low-regulation profile to the high autonomous–introjected profile predicted greater T3 exercise after baseline exercise was controlled. Conversely, movement from intrinsic-dominant motivation to low regulation and from high autonomous–introjected motivation to the amotivated–controlled, low-regulation, mixed intrinsic–controlled, or identified-dominant profiles predicted lower later exercise. These findings extend previous evidence that comparatively autonomous profiles are associated with greater exercise participation or adherence by showing that changes between profiles can also carry behavioral meaning (Emm-Collison et al., 2020; Lindwall et al., 2017; Ruzella et al., 2026; Zheng et al., 2026).

The pattern does not support a simple linear ranking of all profiles. Movement into the high autonomous–introjected profile did not predict significantly greater exercise from every origin, and movement from that profile to the intrinsic-dominant profile did not differ from remaining in it. Retaining strong intrinsic regulation may therefore preserve exercise even when the broader motivational configuration changes. In contrast, transitions involving greater amotivation, weaker intrinsic regulation, or a less favorable overall mixture were generally associated with lower exercise.

The raw-score analysis reproduced the same seven significant contrasts as the primary model, indicating that the overall pattern was not dependent on the log transformation. When T2 exercise was additionally controlled, however, only the transition from low regulation to high autonomous–introjected motivation remained significant. The primary model tested prediction beyond baseline behavior, whereas the stricter model tested whether transitions predicted T3 exercise beyond behavior already reported when the destination profile was assessed. Because T2 exercise may already express part of the motivational transition, attenuation is compatible with motivational and behavioral changes becoming coupled during the transition interval. It could also reflect reciprocal effects or shared unmeasured influences, which the present design cannot distinguish.

The low-regulation-to-high autonomous–introjected transition nevertheless emerged as the clearest behavioral result because it was the most prominent off-diagonal movement in both intervals, was reproduced on the raw GLTEQ scale, and retained prospective value under the stricter adjustment. By contrast, the amotivated–controlled-to-high autonomous–introjected transition had a relatively small posterior expected count and should be interpreted more cautiously. The behavioral hypothesis was therefore supported selectively rather than universally: the implications of motivational movement depended on both the starting configuration and the destination.

### 4.4 Theoretical and practical implications

The findings extend Organismic Integration Theory by representing internalization as movement among multidimensional configurations rather than only as mean-level change in separate regulations. They also extend Basic Psychological Needs Theory by showing that the relevance of need experiences depended on the origin profile: satisfaction was most evident in preserving internalized motivation, whereas frustration was most evident in obstructing internalization from low regulation. Finally, the prospective exercise analyses indicate that motivational mobility can be behaviorally consequential, although attenuation after T2 adjustment suggests close temporal coupling between motivation and behavior. Overall, the evidence supports a dynamic and state-dependent account of exercise internalization, not a single uniform sequence from needs to profile transition to later behavior (Deci and Ryan, 2000; Ryan and Deci, 2017; Vansteenkiste et al., 2020).

Practically, a uniform motivational strategy may be insufficient. For students already in intrinsic-dominant or high autonomous–introjected profiles, the priority may be to preserve need-supportive experiences and prevent deterioration toward low regulation. For students with low regulation, reducing coercive, competence-undermining, or exclusionary experiences may remove barriers to internalization, although the null need findings for movement to the high autonomous–introjected profile indicate that this alone may not be sufficient. Universities could combine meaningful choice with clear structure, offer achievable challenges and informational feedback, connect exercise with personally valued outcomes, and create inclusive opportunities that support enjoyment and social connection. Reviews of self-determination theory–informed interventions indicate generally small-to-moderate improvements in motivational and health-behavior outcomes, supporting these approaches as promising rather than guaranteed (Ntoumanis et al., 2021; Ntoumanis and Moller, 2025). These practices were not experimentally tested here, and the profiles are probabilistic states rather than diagnostic labels for individual students.

### 4.5 Strengths, limitations, and future directions

The study combined a large cohort from six bachelor’s-degree and vocational colleges with strong three-wave retention and two transition intervals. Longitudinal measurement analyses supported comparable interpretation of the measures, and profile definitions were held constant across waves. Posterior probabilities were used rather than treating modal profile assignments as error-free, missing manifest variables were addressed through multiple imputation, sparse comparisons were limited through prespecified expected-count thresholds, and Holm correction controlled familywise error. Baseline-adjusted, T2-adjusted, and raw-scale behavioral models further tested the consistency and boundaries of the findings.

Several limitations qualify the conclusions. First, the convenience sample came from one region of Jiangsu Province. Including six institutions did not establish national representativeness; replication across regions is needed. Second, exercise was assessed using the GLTEQ. Administration of the same established measure across waves and convergence between log-transformed and raw-score analyses supported the consistency of the observed pattern, but self-report remains vulnerable to recall and reporting bias. Future studies should incorporate accelerometry or other device-based measures. Third, the approximately 8-week design was deliberately aligned with the 4-week need-experience reference period and was well suited to examining short-term mobility, but it cannot establish whether transitions persist across semesters or years. The three assessments were also embedded within a single academic term, with T1 administered in October, T2 in November, and T3 in December. Although the assessments were calendar-aligned across all participating colleges and did not coincide with final examinations or major holiday periods, broader time-varying academic influences such as changes in coursework demands, approaching end-of-term responsibilities, weather, or campus exercise opportunities may still have contributed to wave-to-wave changes. Because all participants followed the same calendar schedule, these academic-calendar influences cannot be separated from elapsed-time effects in the present design. Participants were also drawn from all 4 years of study, and the study was not designed to estimate year-specific transition structures; differences in academic demands associated with first-year adjustment, middle years, or later-year study requirements may therefore contribute to heterogeneity not captured by the overall transition model. Longer follow-up, complemented by more frequent assessments across multiple academic periods, would distinguish temporary fluctuations from sustained internalization and permit academic-calendar and year-of-study differences to be examined more directly. Fourth, the observational design does not establish causality. Temporal ordering, adjustment for prior exercise, and the stricter T2-exercise sensitivity analysis reduced some alternative explanations, but reciprocal motivation–behavior effects and unmeasured confounding remain possible. Experimental or quasi-experimental need-supportive interventions are required to test whether transitions can be deliberately changed. Fifth, uncommon transitions remained difficult to estimate. Prespecified count thresholds reduced unstable inference, but excluded very rare movements, and the amotivated–controlled-to-high autonomous–introjected result requires replication because its expected count was small. Larger multisite samples would improve precision. Sixth, posterior-probability weighting limited reliance on potentially error-prone modal classification, but the parameters of the selected latent transition model were treated as fixed. One-step conditional models, bootstrap procedures, or Bayesian estimation could propagate this additional uncertainty. Seventh, the broad need composites were supported by the higher-order measurement model and reduced the number of statistical tests, but may conceal distinct autonomy-, competence-, or relatedness-specific pathways. Eighth, the C-BREQ-2 does not assess integrated regulation, the most fully internalized form of extrinsic motivation in Organismic Integration Theory. Consequently, the latent profiles represent configurations of the five regulation dimensions measured by the C-BREQ-2 rather than the complete theoretical continuum, and labels such as “high autonomous–introjected” refer to the observed identified, intrinsic, and introjected components rather than implying a particular level of integrated regulation. Future longitudinal person-centered research should include an appropriately validated measure of integrated regulation to determine whether this additional dimension produces further profile differentiation or alters observed transition patterns. Ninth, profile enumeration and relative profile stability under RQ1 were exploratory. The study did not specify an *a priori* profile number or a formal criterion for evaluating relative profile stability. Consequently, these features are descriptive rather than confirmatory, and their evidential weight rests on the convergence of model-selection diagnostics, classification quality, random-start reproducibility, and the magnitude and consistency of the estimated transition probabilities across the two intervals. In addition, six profiles were the upper boundary of the candidate set evaluated. Although the retained six-profile solution was supported by the lowest information criteria among the longitudinal models examined, acceptable classification quality and profile size, reproducible estimation, and substantively distinct profile shapes, the present enumeration cannot determine whether models with more than six profiles would provide additional statistical improvement. Conversely, scree-plot inspection involves judgment regarding where diminishing returns become substantively sufficient and does not identify a uniquely correct profile count in these data. The six-profile solution should therefore be interpreted as the best-supported solution within the evaluated candidate set, and broader enumeration and replication in independent samples are warranted to assess the robustness of this degree of profile differentiation. Future studies seeking a confirmatory test of relative profile stability should prespecify an explicit stability criterion or planned contrast. Tenth, the psychological-need analyses estimated origin–destination-specific associations rather than a single omnibus effect on “upward” or “downward” motivational mobility. Aggregating transitions in that way would require a defensible ordinal classification of the empirically derived profiles, which was neither prespecified nor unambiguous for mixed configurations containing both autonomous and controlled regulations. Future studies for which global directional mobility is the primary estimand could prespecify an ordering rule or complementary continuous index before profile estimation and formally evaluate aggregated directional transitions alongside profile-specific effects. Eleventh, the hypotheses and analysis plan were not preregistered. Consequently, although profile enumeration was treated as exploratory and the prospective hypotheses were theory-guided, their temporal precedence and the analytic decisions cannot be independently verified against a time-stamped protocol. This limits the confirmatory strength of the evidence and makes preregistered replication, ideally with the profile structure and follow-up contrasts specified in advance, an important next step. Finally, retention was high, baseline differences were generally small, and maximum likelihood together with multiple imputation used the available information; nevertheless, identified regulation predicted T3 participation, and the missing-at-random assumption cannot be verified. Future replication should combine richer predictors of attrition with sensitivity analyses for departures from that assumption.

## 5 Conclusion

Chinese college students displayed heterogeneous exercise-regulation configurations that were substantially stable but also meaningfully mobile over two 4-week intervals. Need satisfaction was primarily associated with protection against movement from comparatively internalized motivation to low regulation, whereas need frustration was primarily associated with inhibited movement from low regulation toward identified- or intrinsic-dominant motivation. Early transitions also predicted later leisure-time exercise, although movement from low regulation to high autonomous–introjected motivation provided the most consistent evidence across analytic specifications. These findings support viewing exercise motivation as a dynamic, need-sensitive configuration whose behavioral implications depend on the student’s starting state and specific destination.

## Data Availability

The dataset supporting the findings of this study is available in the Open Science Framework (Zhu et al., 2026) at: https://doi.org/10.17605/OSF.IO/PFXZ6.
