# Supplementary material for: Exercise motivation is stable yet mobile: a three-wave latent transition study of psychological-need predictors and subsequent exercise
Source: Front Psychol. 2026 Oct 29;17:1967142. doi: 10.3389/fpsyg.2026.1967142 (PMC13754689; doi:10.3389/fpsyg.2026.1967142)
Supplement: Supplementary file 1 [file Supplementary_file_1.DOCX]

**Supplementary Materials**

**Table S1** *Data-Integrity Checks, Participant Flow, Missingness, and Multiple-Imputation Diagnostics*

**Panel A. Participant flow**

| **Category** | **n** | **% of total** |
| --- | --- | --- |
| Total unique participant IDs | 1,780 | 100.0 |
| T1 retained-data present | 1,780 | 100.0 |
| T2 retained-data present | 1,692 | 95.1 |
| T3 retained-data present | 1,575 | 88.5 |
| All three waves retained-data present | 1,555 | 87.4 |
| Exactly two waves retained-data present | 157 | 8.8 |
| Exactly one wave retained-data present | 68 | 3.8 |
| Intermittent pattern T1+T3 but not T2 | 20 | 1.1 |

**Panel B. Missingness in scored variables by wave**

| **Construct** | **T1 observed** | **T1 missing, n (%)** | **T2 observed** | **T2 missing, n (%)** | **T3 observed** | **T3 missing, n (%)** |
| --- | --- | --- | --- | --- | --- | --- |
| Amotivation | 1,777 | 3 (0.2%) | 1,687 | 93 (5.2%) | 1,570 | 210 (11.8%) |
| External regulation | 1,776 | 4 (0.2%) | 1,690 | 90 (5.1%) | 1,569 | 211 (11.9%) |
| Introjected regulation | 1,777 | 3 (0.2%) | 1,688 | 92 (5.2%) | 1,575 | 205 (11.5%) |
| Identified regulation | 1,777 | 3 (0.2%) | 1,692 | 88 (4.9%) | 1,575 | 205 (11.5%) |
| Intrinsic regulation | 1,773 | 7 (0.4%) | 1,690 | 90 (5.1%) | 1,574 | 206 (11.6%) |
| Autonomy satisfaction | 1,779 | 1 (0.1%) | 1,690 | 90 (5.1%) | 1,574 | 206 (11.6%) |
| Autonomy frustration | 1,779 | 1 (0.1%) | 1,689 | 91 (5.1%) | 1,575 | 205 (11.5%) |
| Relatedness satisfaction | 1,776 | 4 (0.2%) | 1,688 | 92 (5.2%) | 1,571 | 209 (11.7%) |
| Relatedness frustration | 1,776 | 4 (0.2%) | 1,689 | 91 (5.1%) | 1,572 | 208 (11.7%) |
| Competence satisfaction | 1,777 | 3 (0.2%) | 1,686 | 94 (5.3%) | 1,572 | 208 (11.7%) |
| Competence frustration | 1,775 | 5 (0.3%) | 1,682 | 98 (5.5%) | 1,571 | 209 (11.7%) |
| Overall need satisfaction | 1,773 | 7 (0.4%) | 1,682 | 98 (5.5%) | 1,569 | 211 (11.9%) |
| Overall need frustration | 1,774 | 6 (0.3%) | 1,678 | 102 (5.7%) | 1,570 | 210 (11.8%) |
| Weighted GLTEQ | 1,742 | 38 (2.1%) | 1,660 | 120 (6.7%) | 1,547 | 233 (13.1%) |

**Panel C. Missing values before and after multiple imputation**

| **Variable** | **Missing before, n** | **Missing before, %** | **Missing after imputation, n** |
| --- | --- | --- | --- |
| T1 NeedSat | 7 | 0.4% | 0 |
| T1 NeedFrus | 6 | 0.3% | 0 |
| T2 NeedSat | 98 | 5.5% | 0 |
| T2 NeedFrus | 102 | 5.7% | 0 |
| T3 NeedSat | 211 | 11.9% | 0 |
| T3 NeedFrus | 210 | 11.8% | 0 |
| T1 GLTEQ STRENUOUS | 12 | 0.7% | 0 |
| T1 GLTEQ MODERATE | 15 | 0.8% | 0 |
| T1 GLTEQ LIGHT | 14 | 0.8% | 0 |
| T2 GLTEQ STRENUOUS | 98 | 5.5% | 0 |
| T2 GLTEQ MODERATE | 99 | 5.6% | 0 |
| T2 GLTEQ LIGHT | 101 | 5.7% | 0 |
| T3 GLTEQ STRENUOUS | 220 | 12.4% | 0 |
| T3 GLTEQ MODERATE | 214 | 12.0% | 0 |
| T3 GLTEQ LIGHT | 218 | 12.2% | 0 |
| sex | 0 | 0.0% | 0 |
| year of study | 0 | 0.0% | 0 |

*Note.* N = 1,780. Percentages in Panel A use the full T1 cohort as the denominator. GLTEQ = Godin Leisure-Time Exercise Questionnaire. Fifty multiply imputed data sets were created; Panel C reports the first completed data set as an implementation check, and all listed variables had zero remaining missing values. Regulation scores were included as auxiliary variables in the imputation model but latent profile membership was not imputed. The exercise-barrier and sweat-frequency variables were excluded from the final analysis and from the imputation model.

**Table S2** *Complete Descriptive Statistics and Within-Wave Correlations for the Study Variables*

**Panel A. Descriptive statistics**

| **Wave** | **Construct** | **n** | **Missing** | **M** | **SD** | **Median** | **Q1-Q3** | **Min-Max** | **Skew** | **Kurtosis** |
| --- | --- | --- | --- | --- | --- | --- | --- | --- | --- | --- |
| T1 | Amotivation | 1,777 | 3 | 1.45 | 1.01 | 1.25 | 0.50-2.25 | 0.00-4.00 | 0.35 | -0.84 |
| T1 | External regulation | 1,776 | 4 | 1.40 | 0.91 | 1.25 | 0.75-2.00 | 0.00-4.00 | 0.35 | -0.63 |
| T1 | Introjected regulation | 1,777 | 3 | 2.52 | 0.87 | 2.67 | 2.00-3.33 | 0.00-4.00 | -0.21 | -0.68 |
| T1 | Identified regulation | 1,777 | 3 | 2.68 | 0.92 | 2.67 | 2.00-3.33 | 0.00-4.00 | -0.29 | -0.96 |
| T1 | Intrinsic regulation | 1,773 | 7 | 2.65 | 0.98 | 2.75 | 2.00-3.50 | 0.00-4.00 | -0.36 | -0.87 |
| T1 | Autonomy satisfaction | 1,779 | 1 | 3.56 | 0.73 | 3.75 | 3.00-4.00 | 1.50-5.00 | -0.43 | -0.46 |
| T1 | Autonomy frustration | 1,779 | 1 | 2.57 | 0.80 | 2.50 | 2.00-3.00 | 1.00-5.00 | 0.51 | -0.39 |
| T1 | Relatedness satisfaction | 1,776 | 4 | 3.63 | 0.70 | 3.75 | 3.25-4.25 | 1.25-5.00 | -0.35 | -0.32 |
| T1 | Relatedness frustration | 1,776 | 4 | 2.32 | 0.74 | 2.25 | 1.75-2.75 | 1.00-4.50 | 0.51 | -0.29 |
| T1 | Competence satisfaction | 1,777 | 3 | 3.50 | 0.74 | 3.50 | 3.00-4.00 | 1.00-5.00 | -0.31 | -0.50 |
| T1 | Competence frustration | 1,775 | 5 | 2.55 | 0.83 | 2.50 | 2.00-3.00 | 1.00-5.00 | 0.48 | -0.48 |
| T1 | Overall need satisfaction | 1,773 | 7 | 3.56 | 0.61 | 3.67 | 3.08-4.00 | 1.64-4.83 | -0.47 | -0.50 |
| T1 | Overall need frustration | 1,774 | 6 | 2.48 | 0.69 | 2.33 | 1.97-2.92 | 1.08-4.67 | 0.61 | -0.49 |
| T1 | Weighted GLTEQ | 1,742 | 38 | 41.14 | 26.64 | 36.00 | 22.00-55.00 | 0.00-176.00 | 1.24 | 2.33 |
| T1 | log(1 + weighted GLTEQ) | 1,742 | 38 | 3.51 | 0.78 | 3.61 | 3.14-4.03 | 0.00-5.18 | -1.40 | 4.04 |
| T2 | Amotivation | 1,687 | 93 | 1.44 | 1.03 | 1.50 | 0.50-2.25 | 0.00-4.00 | 0.33 | -0.85 |
| T2 | External regulation | 1,690 | 90 | 1.46 | 0.95 | 1.50 | 0.75-2.25 | 0.00-4.00 | 0.32 | -0.75 |
| T2 | Introjected regulation | 1,688 | 92 | 2.54 | 0.91 | 2.67 | 2.00-3.33 | 0.00-4.00 | -0.21 | -0.75 |
| T2 | Identified regulation | 1,692 | 88 | 2.60 | 0.89 | 2.67 | 2.00-3.33 | 0.00-4.00 | -0.19 | -0.96 |
| T2 | Intrinsic regulation | 1,690 | 90 | 2.60 | 0.98 | 2.75 | 1.75-3.50 | 0.00-4.00 | -0.33 | -0.93 |
| T2 | Autonomy satisfaction | 1,690 | 90 | 3.51 | 0.73 | 3.50 | 3.00-4.00 | 1.00-5.00 | -0.41 | -0.39 |
| T2 | Autonomy frustration | 1,689 | 91 | 2.65 | 0.78 | 2.50 | 2.00-3.25 | 1.00-5.00 | 0.43 | -0.43 |
| T2 | Relatedness satisfaction | 1,688 | 92 | 3.64 | 0.70 | 3.75 | 3.25-4.25 | 1.25-5.00 | -0.38 | -0.23 |
| T2 | Relatedness frustration | 1,689 | 91 | 2.36 | 0.75 | 2.25 | 1.75-2.75 | 1.00-5.00 | 0.46 | -0.37 |
| T2 | Competence satisfaction | 1,686 | 94 | 3.52 | 0.77 | 3.50 | 3.00-4.00 | 1.25-5.00 | -0.36 | -0.52 |
| T2 | Competence frustration | 1,682 | 98 | 2.62 | 0.81 | 2.50 | 2.00-3.25 | 1.00-5.00 | 0.41 | -0.42 |
| T2 | Overall need satisfaction | 1,682 | 98 | 3.56 | 0.62 | 3.67 | 3.08-4.00 | 1.50-4.92 | -0.46 | -0.44 |
| T2 | Overall need frustration | 1,678 | 102 | 2.54 | 0.68 | 2.42 | 2.00-3.08 | 1.17-4.50 | 0.54 | -0.61 |
| T2 | Weighted GLTEQ | 1,660 | 120 | 41.03 | 33.79 | 32.00 | 17.00-54.00 | 0.00-272.00 | 1.95 | 5.63 |
| T2 | log(1 + weighted GLTEQ) | 1,660 | 120 | 3.43 | 0.84 | 3.50 | 2.89-4.01 | 0.00-5.61 | -0.66 | 1.41 |
| T3 | Amotivation | 1,570 | 210 | 1.50 | 1.02 | 1.50 | 0.75-2.25 | 0.00-4.00 | 0.39 | -0.74 |
| T3 | External regulation | 1,569 | 211 | 1.50 | 0.92 | 1.50 | 0.75-2.25 | 0.00-4.00 | 0.24 | -0.79 |
| T3 | Introjected regulation | 1,575 | 205 | 2.48 | 0.86 | 2.33 | 2.00-3.00 | 0.00-4.00 | -0.18 | -0.65 |
| T3 | Identified regulation | 1,575 | 205 | 2.62 | 0.93 | 2.67 | 2.00-3.33 | 0.33-4.00 | -0.26 | -0.95 |
| T3 | Intrinsic regulation | 1,574 | 206 | 2.64 | 0.96 | 2.75 | 2.00-3.50 | 0.00-4.00 | -0.33 | -0.96 |
| T3 | Autonomy satisfaction | 1,574 | 206 | 3.52 | 0.73 | 3.58 | 3.00-4.00 | 1.25-5.00 | -0.34 | -0.55 |
| T3 | Autonomy frustration | 1,575 | 205 | 2.54 | 0.80 | 2.50 | 2.00-3.00 | 1.00-5.00 | 0.57 | -0.34 |
| T3 | Relatedness satisfaction | 1,571 | 209 | 3.62 | 0.70 | 3.75 | 3.25-4.25 | 1.25-5.00 | -0.32 | -0.31 |
| T3 | Relatedness frustration | 1,572 | 208 | 2.35 | 0.74 | 2.25 | 1.75-2.75 | 1.00-4.50 | 0.46 | -0.52 |
| T3 | Competence satisfaction | 1,572 | 208 | 3.49 | 0.75 | 3.50 | 3.00-4.00 | 1.25-5.00 | -0.38 | -0.30 |
| T3 | Competence frustration | 1,571 | 209 | 2.54 | 0.83 | 2.50 | 2.00-3.25 | 1.00-5.00 | 0.40 | -0.52 |
| T3 | Overall need satisfaction | 1,569 | 211 | 3.54 | 0.61 | 3.67 | 3.17-4.00 | 1.75-5.00 | -0.43 | -0.49 |
| T3 | Overall need frustration | 1,570 | 210 | 2.48 | 0.69 | 2.33 | 1.92-3.00 | 1.25-4.42 | 0.57 | -0.62 |
| T3 | Weighted GLTEQ | 1,547 | 233 | 42.51 | 38.41 | 32.00 | 18.00-53.00 | 0.00-395.00 | 2.56 | 10.48 |
| T3 | log(1 + weighted GLTEQ) | 1,547 | 233 | 3.44 | 0.87 | 3.50 | 2.94-3.99 | 0.00-5.98 | -0.70 | 1.95 |

**Panel B. T1 within-wave correlations**

| **Variable** | **1** | **2** | **3** | **4** | **5** | **6** | **7** | **8** |
| --- | --- | --- | --- | --- | --- | --- | --- | --- |
| 1. Amotivation | 1.00 | — | — | — | — | — | — | — |
| 2. External regulation | 0.55 | 1.00 | — | — | — | — | — | — |
| 3. Introjected regulation | -0.27 | -0.25 | 1.00 | — | — | — | — | — |
| 4. Identified regulation | -0.38 | -0.30 | 0.42 | 1.00 | — | — | — | — |
| 5. Intrinsic regulation | -0.42 | -0.35 | 0.45 | 0.51 | 1.00 | — | — | — |
| 6. Need satisfaction | -0.42 | -0.35 | 0.48 | 0.58 | 0.59 | 1.00 | — | — |
| 7. Need frustration | 0.58 | 0.52 | -0.39 | -0.51 | -0.55 | -0.60 | 1.00 | — |
| 8. log(1 + weighted GLTEQ) | -0.24 | -0.20 | 0.23 | 0.28 | 0.30 | 0.33 | -0.35 | 1.00 |

**Panel C. T2 within-wave correlations**

| **Variable** | **1** | **2** | **3** | **4** | **5** | **6** | **7** | **8** |
| --- | --- | --- | --- | --- | --- | --- | --- | --- |
| 1. Amotivation | 1.00 | — | — | — | — | — | — | — |
| 2. External regulation | 0.58 | 1.00 | — | — | — | — | — | — |
| 3. Introjected regulation | -0.32 | -0.27 | 1.00 | — | — | — | — | — |
| 4. Identified regulation | -0.38 | -0.30 | 0.44 | 1.00 | — | — | — | — |
| 5. Intrinsic regulation | -0.40 | -0.37 | 0.46 | 0.50 | 1.00 | — | — | — |
| 6. Need satisfaction | -0.43 | -0.37 | 0.45 | 0.56 | 0.58 | 1.00 | — | — |
| 7. Need frustration | 0.58 | 0.54 | -0.40 | -0.46 | -0.54 | -0.59 | 1.00 | — |
| 8. log(1 + weighted GLTEQ) | -0.27 | -0.23 | 0.22 | 0.28 | 0.33 | 0.37 | -0.39 | 1.00 |

**Panel D. T3 within-wave correlations**

| **Variable** | **1** | **2** | **3** | **4** | **5** | **6** | **7** | **8** |
| --- | --- | --- | --- | --- | --- | --- | --- | --- |
| 1. Amotivation | 1.00 | — | — | — | — | — | — | — |
| 2. External regulation | 0.56 | 1.00 | — | — | — | — | — | — |
| 3. Introjected regulation | -0.26 | -0.21 | 1.00 | — | — | — | — | — |
| 4. Identified regulation | -0.38 | -0.31 | 0.44 | 1.00 | — | — | — | — |
| 5. Intrinsic regulation | -0.43 | -0.36 | 0.44 | 0.52 | 1.00 | — | — | — |
| 6. Need satisfaction | -0.40 | -0.36 | 0.46 | 0.57 | 0.61 | 1.00 | — | — |
| 7. Need frustration | 0.55 | 0.50 | -0.37 | -0.50 | -0.56 | -0.59 | 1.00 | — |
| 8. log(1 + weighted GLTEQ) | -0.26 | -0.23 | 0.23 | 0.30 | 0.34 | 0.35 | -0.39 | 1.00 |

*Note.* M = mean; SD = standard deviation; Q1 = first quartile; Q3 = third quartile; GLTEQ = Godin Leisure-Time Exercise Questionnaire. Pearson correlations were calculated using available variable pairs. The correlation matrices use log(1 + weighted GLTEQ), whereas the descriptive panel reports both the raw weighted GLTEQ score and its log transformation. Weighted GLTEQ = 9(strenuous sessions) + 5(moderate sessions) + 3(light sessions).

**Table S3** *Baseline Differences by T3 Participation and Logistic Regression Predicting T3 Retention*

**Panel A. Standardized baseline differences by T3 participation**

| **Variable/level** | **T3 absent n** | **T3 absent M or %** | **T3 present n** | **T3 present M or %** | **SMD** |
| --- | --- | --- | --- | --- | --- |
| Amotivation | 205 | 1.60 | 1,572 | 1.43 | -0.175 |
| External regulation | 204 | 1.44 | 1,572 | 1.40 | -0.049 |
| Introjected regulation | 205 | 2.41 | 1,572 | 2.53 | 0.141 |
| Identified regulation | 205 | 2.51 | 1,572 | 2.70 | 0.205 |
| Intrinsic regulation | 205 | 2.52 | 1,568 | 2.67 | 0.148 |
| Need satisfaction | 205 | 3.49 | 1,568 | 3.57 | 0.143 |
| Need frustration | 205 | 2.59 | 1,569 | 2.47 | -0.180 |
| log(1 + weighted GLTEQ) | 198 | 3.41 | 1,544 | 3.52 | 0.146 |

**Panel B. Logistic regression predicting T3 participation**

| **Predictor** | **B** | **Robust SE** | **z** | **OR [95% CI]** | **p** | **Analysis n** |
| --- | --- | --- | --- | --- | --- | --- |
| Intercept | 1.296 | 0.849 | 1.53 | 3.66 [0.69, 19.32] | .127 | 1,725 |
| Amotivation | -0.109 | 0.098 | -1.12 | 0.90 [0.74, 1.09] | .264 | 1,725 |
| External regulation | 0.109 | 0.101 | 1.08 | 1.12 [0.92, 1.36] | .278 | 1,725 |
| Introjected regulation | 0.062 | 0.104 | 0.59 | 1.06 [0.87, 1.30] | .553 | 1,725 |
| Identified regulation | 0.222 | 0.104 | 2.15 | 1.25 [1.02, 1.53] | .032 | 1,725 |
| Intrinsic regulation | 0.012 | 0.102 | 0.11 | 1.01 [0.83, 1.24] | .909 | 1,725 |
| Need satisfaction | -0.173 | 0.182 | -0.95 | 0.84 [0.59, 1.20] | .341 | 1,725 |
| Need frustration | -0.057 | 0.166 | -0.34 | 0.95 [0.68, 1.31] | .734 | 1,725 |
| log(1 + weighted GLTEQ) | 0.122 | 0.096 | 1.28 | 1.13 [0.94, 1.36] | .202 | 1,725 |

*Note.* T3 present indicates that retained study data were available at T3. For numerical variables, Panel A reports means; for categorical levels, it reports proportions. The SMD is oriented as T3 present minus T3 absent, so a positive value indicates a higher mean or proportion among participants retained at T3. The logistic regression outcome was T3 participation (1 = present). OR = odds ratio; CI = confidence interval; SMD = standardized mean difference. P values are unadjusted and the attrition analyses were descriptive.

**Table S4** *Internal Consistency, Composite Reliability, and Standardized Confirmatory Factor-Analysis Loadings*

**Panel A. Internal consistency and composite reliability by wave**

| **Scale** | **Items** | **T1 complete n** | **T1 alpha** | **T1 omega** | **T2 complete n** | **T2 alpha** | **T2 omega** | **T3 complete n** | **T3 alpha** | **T3 omega** |
| --- | --- | --- | --- | --- | --- | --- | --- | --- | --- | --- |
| Amotivation | 4 | 1,738 | 0.869 | 0.872 | 1,662 | 0.880 | 0.885 | 1,546 | 0.866 | 0.868 |
| External regulation | 4 | 1,744 | 0.813 | 0.811 | 1,664 | 0.834 | 0.833 | 1,554 | 0.804 | 0.803 |
| Introjected regulation | 3 | 1,746 | 0.771 | 0.775 | 1,669 | 0.831 | 0.832 | 1,556 | 0.759 | 0.759 |
| Identified regulation | 3 | 1,758 | 0.755 | 0.752 | 1,678 | 0.683 | 0.681 | 1,544 | 0.739 | 0.741 |
| Intrinsic regulation | 4 | 1,739 | 0.844 | 0.847 | 1,657 | 0.846 | 0.847 | 1,547 | 0.847 | 0.849 |
| Autonomy satisfaction | 4 | 1,729 | 0.708 | 0.714 | 1,644 | 0.700 | 0.712 | 1,527 | 0.724 | 0.728 |
| Autonomy frustration | 4 | 1,741 | 0.773 | 0.770 | 1,649 | 0.737 | 0.740 | 1,539 | 0.778 | 0.775 |
| Relatedness satisfaction | 4 | 1,718 | 0.698 | 0.696 | 1,634 | 0.704 | 0.692 | 1,537 | 0.720 | 0.707 |
| Relatedness frustration | 4 | 1,730 | 0.765 | 0.757 | 1,649 | 0.772 | 0.764 | 1,529 | 0.767 | 0.763 |
| Competence satisfaction | 4 | 1,713 | 0.752 | 0.758 | 1,650 | 0.771 | 0.776 | 1,528 | 0.758 | 0.765 |
| Competence frustration | 4 | 1,725 | 0.805 | 0.799 | 1,650 | 0.775 | 0.772 | 1,536 | 0.816 | 0.815 |
| Overall need satisfaction | 12 | 1,635 | 0.856 | — | 1,578 | 0.857 | — | 1,468 | 0.860 | — |
| Overall need frustration | 12 | 1,670 | 0.893 | — | 1,599 | 0.882 | — | 1,484 | 0.896 | — |

**Panel B. Standardized C-BREQ-2 item loadings**

| **Factor** | **Item code** | **T1 loading** | **T2 loading** | **T3 loading** |
| --- | --- | --- | --- | --- |
| Amotivation | BREQ_AMO1 | 0.828 | 0.865 | 0.814 |
| Amotivation | BREQ_AMO2 | 0.787 | 0.842 | 0.809 |
| Amotivation | BREQ_AMO3 | 0.866 | 0.883 | 0.857 |
| Amotivation | BREQ_AMO4 | 0.820 | 0.774 | 0.799 |
| External regulation | BREQ_EXT1 | 0.768 | 0.765 | 0.781 |
| External regulation | BREQ_EXT2 | 0.692 | 0.745 | 0.672 |
| External regulation | BREQ_EXT3 | 0.847 | 0.856 | 0.814 |
| External regulation | BREQ_EXT4 | 0.714 | 0.753 | 0.708 |
| Introjected regulation | BREQ_INTJ1 | 0.744 | 0.848 | 0.760 |
| Introjected regulation | BREQ_INTJ2 | 0.836 | 0.877 | 0.772 |
| Introjected regulation | BREQ_INTJ3 | 0.713 | 0.760 | 0.721 |
| Identified regulation | BREQ_IDEN1 | 0.747 | 0.690 | 0.724 |
| Identified regulation | BREQ_IDEN2 | 0.714 | 0.621 | 0.670 |
| Identified regulation | BREQ_IDEN3 | 0.778 | 0.728 | 0.799 |
| Intrinsic regulation | BREQ_INTR1 | 0.836 | 0.828 | 0.795 |
| Intrinsic regulation | BREQ_INTR2 | 0.756 | 0.750 | 0.779 |
| Intrinsic regulation | BREQ_INTR3 | 0.863 | 0.854 | 0.883 |
| Intrinsic regulation | BREQ_INTR4 | 0.723 | 0.748 | 0.729 |

**Panel C. Standardized BPNSFS item loadings**

| **Factor** | **Item code** | **T1 loading** | **T2 loading** | **T3 loading** |
| --- | --- | --- | --- | --- |
| Autonomy satisfaction | BPNS01 | 0.684 | 0.642 | 0.678 |
| Autonomy satisfaction | BPNS07 | 0.574 | 0.579 | 0.580 |
| Autonomy satisfaction | BPNS13 | 0.734 | 0.807 | 0.810 |
| Autonomy satisfaction | BPNS19 | 0.611 | 0.563 | 0.596 |
| Autonomy frustration | BPNS02 | 0.778 | 0.766 | 0.736 |
| Autonomy frustration | BPNS08 | 0.602 | 0.614 | 0.652 |
| Autonomy frustration | BPNS14 | 0.758 | 0.689 | 0.794 |
| Autonomy frustration | BPNS20 | 0.680 | 0.615 | 0.661 |
| Relatedness satisfaction | BPNS03 | 0.698 | 0.673 | 0.758 |
| Relatedness satisfaction | BPNS09 | 0.542 | 0.547 | 0.471 |
| Relatedness satisfaction | BPNS15 | 0.756 | 0.768 | 0.806 |
| Relatedness satisfaction | BPNS21 | 0.575 | 0.587 | 0.626 |
| Relatedness frustration | BPNS04 | 0.771 | 0.797 | 0.758 |
| Relatedness frustration | BPNS10 | 0.592 | 0.612 | 0.582 |
| Relatedness frustration | BPNS16 | 0.801 | 0.762 | 0.760 |
| Relatedness frustration | BPNS22 | 0.658 | 0.673 | 0.728 |
| Competence satisfaction | BPNS05 | 0.744 | 0.742 | 0.804 |
| Competence satisfaction | BPNS11 | 0.598 | 0.548 | 0.573 |
| Competence satisfaction | BPNS17 | 0.771 | 0.865 | 0.768 |
| Competence satisfaction | BPNS23 | 0.669 | 0.703 | 0.664 |
| Competence frustration | BPNS06 | 0.815 | 0.767 | 0.825 |
| Competence frustration | BPNS12 | 0.642 | 0.645 | 0.678 |
| Competence frustration | BPNS18 | 0.814 | 0.771 | 0.853 |
| Competence frustration | BPNS24 | 0.696 | 0.653 | 0.680 |

**Panel D. Standardized higher-order BPNSFS loadings**

| **Higher-order factor** | **First-order factor** | **T1 loading** | **T2 loading** | **T3 loading** |
| --- | --- | --- | --- | --- |
| Need satisfaction | Autonomy satisfaction | 0.929 | 0.904 | 0.948 |
| Need satisfaction | Competence satisfaction | 0.887 | 0.924 | 0.857 |
| Need satisfaction | Relatedness satisfaction | 0.828 | 0.792 | 0.817 |
| Need frustration | Autonomy frustration | 0.915 | 0.948 | 0.941 |
| Need frustration | Competence frustration | 0.896 | 0.886 | 0.899 |
| Need frustration | Relatedness frustration | 0.894 | 0.866 | 0.871 |

*Note.* Alpha is Cronbach's raw alpha. Omega is model-based composite reliability from the wave-specific correlated-factor CFA. Omega was not calculated for the 12-item broad need composites; support for those composites was instead evaluated with the higher-order BPNSFS model. Item loadings in Panels B and C come from the correlated first-order models. Panel D reports the standardized loadings of the three satisfaction dimensions on need satisfaction and the three frustration dimensions on need frustration. All displayed standardized loadings were statistically significant at p < .001.

**Table S5** *Full Confirmatory Factor-Analysis Fit and Longitudinal Measurement-Invariance Results*

**Panel A. Wave-specific confirmatory factor analyses**

| **Instrument** | **Model** | **Wave** | **n** | **Scaled chi-square (df)** | **p** | **CFI** | **TLI** | **RMSEA [90% CI]** | **SRMR** |
| --- | --- | --- | --- | --- | --- | --- | --- | --- | --- |
| BPNS | Six correlated factors | T1 | 1,780 | 455.77 (237) | <.001 | 0.993 | 0.992 | 0.023 [0.020, 0.026] | 0.024 |
| BPNS | Six first-order + two higher-order factors | T1 | 1,780 | 592.84 (245) | <.001 | 0.989 | 0.988 | 0.028 [0.025, 0.031] | 0.028 |
| BPNS | Six correlated factors | T2 | 1,692 | 572.27 (237) | <.001 | 0.989 | 0.987 | 0.029 [0.026, 0.032] | 0.028 |
| BPNS | Six first-order + two higher-order factors | T2 | 1,692 | 742.91 (245) | <.001 | 0.983 | 0.981 | 0.035 [0.032, 0.038] | 0.033 |
| BPNS | Six correlated factors | T3 | 1,575 | 437.69 (237) | <.001 | 0.994 | 0.992 | 0.023 [0.020, 0.027] | 0.024 |
| BPNS | Six first-order + two higher-order factors | T3 | 1,575 | 584.40 (245) | <.001 | 0.989 | 0.988 | 0.030 [0.027, 0.033] | 0.030 |
| BREQ | Five correlated factors | T1 | 1,780 | 367.55 (125) | <.001 | 0.991 | 0.989 | 0.033 [0.029, 0.037] | 0.027 |
| BREQ | Five correlated factors | T2 | 1,692 | 321.34 (125) | <.001 | 0.993 | 0.992 | 0.030 [0.026, 0.035] | 0.025 |
| BREQ | Five correlated factors | T3 | 1,575 | 330.04 (125) | <.001 | 0.991 | 0.989 | 0.032 [0.028, 0.037] | 0.027 |

**Panel B. Longitudinal invariance model fit**

| **Instrument** | **Invariance model** | **Scaled chi-square (df)** | **p** | **CFI** | **TLI** | **RMSEA [90% CI]** | **SRMR** | **Delta CFI** | **Delta RMSEA** |
| --- | --- | --- | --- | --- | --- | --- | --- | --- | --- |
| BREQ | Configural | 1800.61 (1218) | <.001 | 0.992 | 0.991 | 0.016 [0.015, 0.018] | 0.023 | — | — |
| BREQ | Threshold | 1876.26 (1290) | <.001 | 0.992 | 0.991 | 0.016 [0.014, 0.018] | 0.023 | -0.00005 | -0.00041 |
| BREQ | Threshold + loading | 1886.61 (1316) | <.001 | 0.992 | 0.992 | 0.016 [0.014, 0.017] | 0.023 | 0.00021 | -0.00037 |
| BPNS | Configural | 2840.72 (2259) | <.001 | 0.993 | 0.992 | 0.012 [0.011, 0.013] | 0.023 | — | — |
| BPNS | Threshold | 2942.16 (2355) | <.001 | 0.993 | 0.992 | 0.012 [0.010, 0.013] | 0.023 | -0.00006 | -0.00019 |
| BPNS | Threshold + loading | 3004.07 (2391) | <.001 | 0.993 | 0.992 | 0.012 [0.011, 0.013] | 0.023 | -0.00031 | 0.00017 |

**Panel C. Scaled chi-square difference tests**

| **Instrument** | **Comparison** | **Delta chi-square** | **Delta df** | **p** |
| --- | --- | --- | --- | --- |
| BREQ | Configural vs threshold | 69.47 | 72 | .563 |
| BREQ | Threshold vs threshold + loading | 34.85 | 26 | .115 |
| BPNS | Configural vs threshold | 105.17 | 96 | .245 |
| BPNS | Threshold vs threshold + loading | 65.94 | 36 | .002 |

*Note.* C-BREQ-2 = Chinese Behavioral Regulation in Exercise Questionnaire-2; BPNSFS = Basic Psychological Need Satisfaction and Frustration Scale; CFI = comparative fit index; TLI = Tucker-Lewis index; RMSEA = root mean square error of approximation; SRMR = standardized root mean square residual. Models used WLSMV with ordered indicators and theta parameterization. Delta values compare each model with the immediately preceding, less constrained model. The BPNSFS threshold-to-loading scaled difference test was statistically significant, but changes in CFI and RMSEA were very small.

**Table S6** *Supportive Cross-Sectional Latent Profile Analyses by Wave*

**Panel A. Profile-enumeration fit statistics**

| **Wave** | **Covariance** | **K** | **n** | **Log-likelihood** | **Parameters** | **AIC** | **BIC** | **ICL** | **Entropy** | **Smallest %** |
| --- | --- | --- | --- | --- | --- | --- | --- | --- | --- | --- |
| T1 | EEI | 1 | 1,767 | -11965.26 | 10 | 23950.52 | 24005.29 | -24005.29 | 1.000 | 100.0 |
| T1 | EEI | 2 | 1,767 | -10853.32 | 16 | 21738.65 | 21826.28 | -22022.04 | 0.838 | 42.0 |
| T1 | EEI | 3 | 1,767 | -10787.40 | 22 | 21618.81 | 21739.30 | -22078.21 | 0.812 | 8.9 |
| T1 | EEI | 4 | 1,767 | -10649.88 | 28 | 21355.76 | 21509.11 | -22019.90 | 0.780 | 10.9 |
| T1 | EEI | 5 | 1,767 | -10651.93 | 34 | 21371.85 | 21558.07 | -22591.07 | 0.663 | 11.3 |
| **T1** | **EEI** | **6** | **1,767** | **-10555.58** | **40** | **21191.17** | **21410.25** | **-22253.69** | **0.731** | **8.6** |
| T1 | EEE | 1 | 1,767 | -10889.40 | 20 | 21818.81 | 21928.35 | -21928.35 | 1.000 | 100.0 |
| T1 | EEE | 2 | 1,767 | -10669.47 | 26 | 21390.94 | 21533.34 | -21706.78 | 0.851 | 40.9 |
| T1 | EEE | 3 | 1,767 | -10632.12 | 32 | 21328.25 | 21503.51 | -21856.70 | 0.808 | 8.7 |
| T1 | EEE | 4 | 1,767 | -10631.78 | 38 | 21339.56 | 21547.69 | -22737.31 | 0.608 | 8.7 |
| T1 | EEE | 5 | 1,767 | -10516.45 | 44 | 21120.89 | 21361.88 | -22331.82 | 0.692 | 12.7 |
| **T1** | **EEE** | **6** | **1,767** | **-10481.86** | **50** | **21063.72** | **21337.57** | **-22296.70** | **0.709** | **7.1** |
| T2 | EEI | 1 | 1,681 | -11497.06 | 10 | 23014.12 | 23068.39 | -23068.39 | 1.000 | 100.0 |
| T2 | EEI | 2 | 1,681 | -10379.97 | 16 | 20791.93 | 20878.77 | -21045.07 | 0.850 | 44.8 |
| T2 | EEI | 3 | 1,681 | -10282.51 | 22 | 20609.02 | 20728.41 | -21071.26 | 0.809 | 11.5 |
| T2 | EEI | 4 | 1,681 | -10207.92 | 28 | 20471.84 | 20623.80 | -21140.83 | 0.775 | 8.3 |
| T2 | EEI | 5 | 1,681 | -10155.21 | 34 | 20378.43 | 20562.95 | -21206.14 | 0.758 | 5.7 |
| **T2** | **EEI** | **6** | **1,681** | **-10128.28** | **40** | **20336.57** | **20553.65** | **-21287.96** | **0.749** | **5.8** |
| T2 | EEE | 1 | 1,681 | -10413.47 | 20 | 20866.94 | 20975.48 | -20975.48 | 1.000 | 100.0 |
| T2 | EEE | 2 | 1,681 | -10195.69 | 26 | 20443.37 | 20584.48 | -20733.99 | 0.860 | 43.2 |
| T2 | EEE | 3 | 1,681 | -10162.25 | 32 | 20388.50 | 20562.17 | -20928.69 | 0.786 | 11.2 |
| **T2** | **EEE** | **4** | **1,681** | **-10091.52** | **38** | **20259.05** | **20465.28** | **-20897.51** | **0.805** | **12.0** |
| T2 | EEE | 5 | 1,681 | -10073.99 | 44 | 20235.97 | 20474.77 | -20992.59 | 0.796 | 5.3 |
| T2 | EEE | 6 | 1,681 | -10092.14 | 50 | 20284.28 | 20555.64 | -21345.97 | 0.729 | 3.8 |
| T3 | EEI | 1 | 1,567 | -10593.70 | 10 | 21207.39 | 21260.96 | -21260.96 | 1.000 | 100.0 |
| T3 | EEI | 2 | 1,567 | -9607.69 | 16 | 19247.38 | 19333.09 | -19501.73 | 0.840 | 42.4 |
| T3 | EEI | 3 | 1,567 | -9515.98 | 22 | 19075.95 | 19193.80 | -19536.07 | 0.796 | 12.5 |
| T3 | EEI | 4 | 1,567 | -9406.31 | 28 | 18868.62 | 19018.62 | -19487.79 | 0.780 | 13.7 |
| T3 | EEI | 5 | 1,567 | -9390.90 | 34 | 18849.79 | 19031.93 | -19715.48 | 0.725 | 9.9 |
| **T3** | **EEI** | **6** | **1,567** | **-9311.44** | **40** | **18702.88** | **18917.16** | **-19475.90** | **0.790** | **6.3** |
| T3 | EEE | 1 | 1,567 | -9603.25 | 20 | 19246.50 | 19353.64 | -19353.64 | 1.000 | 100.0 |
| T3 | EEE | 2 | 1,567 | -9427.93 | 26 | 18907.87 | 19047.15 | -19211.26 | 0.838 | 41.3 |
| T3 | EEE | 3 | 1,567 | -9389.45 | 32 | 18842.90 | 19014.32 | -19322.41 | 0.807 | 8.9 |
| T3 | EEE | 4 | 1,567 | -9378.50 | 38 | 18833.00 | 19036.56 | -19604.98 | 0.728 | 7.9 |
| T3 | EEE | 5 | 1,567 | -9328.79 | 44 | 18745.58 | 18981.29 | -19645.03 | 0.729 | 9.2 |
| **T3** | **EEE** | **6** | **1,567** | **-9300.34** | **50** | **18700.68** | **18968.52** | **-19658.83** | **0.740** | **4.4** |

**Panel B. Six-profile wave-specific means and classification quality**

| **Wave** | **Covariance** | **Profile** | **AMO** | **EXT** | **INTJ** | **IDEN** | **INTR** | **Posterior %** | **Modal n** | **Avg posterior p** |
| --- | --- | --- | --- | --- | --- | --- | --- | --- | --- | --- |
| T1 | EEE | P1 | 2.33 | 2.05 | 1.83 | 1.69 | 1.61 | 26.5 | 482 | 0.909 |
| T1 | EEE | P2 | 2.18 | 2.28 | 2.84 | 2.31 | 3.08 | 7.1 | 113 | 0.705 |
| T1 | EEE | P3 | 1.72 | 1.85 | 2.24 | 3.14 | 1.76 | 11.7 | 210 | 0.814 |
| T1 | EEE | P4 | 1.14 | 0.90 | 2.35 | 1.85 | 3.05 | 10.7 | 186 | 0.791 |
| T1 | EEE | P5 | 0.67 | 0.76 | 3.16 | 3.31 | 3.08 | 12.1 | 74 | 0.540 |
| T1 | EEE | P6 | 0.86 | 0.92 | 2.92 | 3.43 | 3.44 | 32.0 | 702 | 0.728 |
| T1 | EEI | P1 | 2.32 | 2.04 | 1.81 | 1.69 | 1.58 | 25.9 | 466 | 0.912 |
| T1 | EEI | P2 | 2.46 | 2.37 | 2.73 | 2.61 | 2.92 | 10.5 | 182 | 0.782 |
| T1 | EEI | P3 | 1.53 | 1.70 | 2.15 | 3.13 | 1.66 | 8.6 | 152 | 0.767 |
| T1 | EEI | P4 | 1.03 | 0.90 | 2.32 | 1.83 | 2.94 | 10.9 | 196 | 0.794 |
| T1 | EEI | P5 | 0.55 | 0.61 | 3.06 | 3.14 | 2.93 | 12.5 | 155 | 0.642 |
| T1 | EEI | P6 | 0.88 | 0.97 | 2.97 | 3.48 | 3.50 | 31.5 | 616 | 0.797 |
| T2 | EEE | P1 | 2.23 | 1.54 | 1.91 | 1.92 | 1.38 | 18.0 | 299 | 0.836 |
| T2 | EEE | P2 | 2.09 | 2.54 | 1.94 | 2.17 | 1.88 | 22.5 | 402 | 0.810 |
| T2 | EEE | P3 | 1.78 | 2.72 | 2.84 | 2.24 | 3.11 | 3.8 | 47 | 0.745 |
| T2 | EEE | P4 | 1.46 | 0.89 | 2.42 | 1.92 | 3.05 | 7.8 | 113 | 0.691 |
| T2 | EEE | P5 | 1.09 | 1.78 | 2.98 | 3.32 | 3.44 | 10.4 | 153 | 0.700 |
| T2 | EEE | P6 | 0.73 | 0.68 | 3.07 | 3.17 | 3.23 | 37.5 | 667 | 0.851 |
| T2 | EEI | P1 | 2.81 | 2.75 | 2.18 | 2.06 | 1.93 | 12.2 | 187 | 0.774 |
| T2 | EEI | P2 | 1.99 | 1.90 | 1.81 | 1.88 | 1.53 | 24.5 | 438 | 0.808 |
| T2 | EEI | P3 | 1.77 | 1.31 | 2.71 | 1.84 | 3.17 | 5.8 | 86 | 0.690 |
| T2 | EEI | P4 | 1.83 | 2.01 | 2.74 | 3.20 | 3.08 | 12.4 | 204 | 0.746 |
| T2 | EEI | P5 | 0.56 | 0.67 | 1.94 | 2.04 | 2.37 | 6.3 | 91 | 0.752 |
| T2 | EEI | P6 | 0.63 | 0.75 | 3.11 | 3.25 | 3.27 | 38.8 | 675 | 0.903 |
| T3 | EEE | P1 | 2.50 | 2.25 | 1.92 | 1.76 | 1.72 | 27.6 | 464 | 0.858 |
| T3 | EEE | P2 | 1.29 | 1.75 | 1.59 | 1.96 | 1.56 | 4.4 | 39 | 0.598 |
| T3 | EEE | P3 | 1.81 | 1.77 | 2.42 | 3.21 | 1.86 | 9.1 | 135 | 0.776 |
| T3 | EEE | P4 | 1.44 | 1.41 | 2.46 | 1.91 | 3.13 | 14.8 | 235 | 0.806 |
| T3 | EEE | P5 | 0.79 | 0.96 | 3.12 | 3.34 | 3.31 | 36.5 | 620 | 0.849 |
| T3 | EEE | P6 | 1.13 | 1.06 | 2.11 | 3.36 | 3.40 | 7.7 | 74 | 0.666 |
| T3 | EEI | P1 | 2.51 | 2.30 | 1.92 | 1.72 | 1.64 | 21.8 | 358 | 0.839 |
| T3 | EEI | P2 | 1.44 | 1.46 | 1.44 | 1.55 | 1.36 | 6.3 | 88 | 0.745 |
| T3 | EEI | P3 | 2.80 | 2.46 | 2.66 | 2.58 | 2.84 | 9.1 | 131 | 0.805 |
| T3 | EEI | P4 | 1.57 | 1.66 | 2.34 | 3.20 | 1.92 | 8.3 | 126 | 0.743 |
| T3 | EEI | P5 | 1.03 | 1.10 | 2.36 | 1.90 | 2.96 | 13.0 | 193 | 0.824 |
| T3 | EEI | P6 | 0.83 | 0.97 | 2.97 | 3.38 | 3.37 | 41.5 | 671 | 0.921 |

*Note.* K = number of profiles; AIC = Akaike information criterion; BIC = Bayesian information criterion; ICL = integrated completed likelihood as returned by mclust; AMO = amotivation; EXT = external regulation; INTJ = introjected regulation; IDEN = identified regulation; INTR = intrinsic regulation. EEI represents an equal diagonal covariance structure; EEE represents an equal unrestricted covariance structure. Lower AIC/BIC and higher ICL values indicate better relative fit under the reported conventions. The minimum BIC within each wave and covariance specification is bolded. Cross-sectional P1-P6 labels were ordered within each solution using the internalization-ordering index and do not necessarily correspond one-to-one with the final longitudinal P1-P6 labels.

**Table S7** *Latent Transition Analysis Enumeration, Random-Start Diagnostics, and Focused Five- Versus Six-Profile Confirmation*

**Panel A. Longitudinal profile enumeration**

| **K** | **Log-likelihood** | **Parameters** | **AIC** | **BIC** | **Adjusted BIC** | **Entropy** | **Smallest %** | **Initial starts** | **Focused starts** | **Starts within 0.01** |
| --- | --- | --- | --- | --- | --- | --- | --- | --- | --- | --- |
| 1 | -31084.46 | 20 | 62208.91 | 62318.60 | 62255.06 | 1.000 | 100.0 | 1 | — | 1 |
| 2 | -29855.02 | 30 | 59770.04 | 59934.57 | 59839.27 | 0.850 | 40.6 | 20 | — | 20 |
| 3 | -29526.46 | 44 | 59140.92 | 59382.23 | 59242.45 | 0.828 | 13.6 | 20 | — | 19 |
| 4 | -29081.03 | 62 | 58286.06 | 58626.09 | 58429.12 | 0.832 | 11.7 | 20 | — | 20 |
| 5 | -28930.81 | 84 | 58029.63 | 58490.31 | 58223.45 | 0.835 | 5.6 | 20 | 100 | 58 |
| **6** | **-28809.49** | **110** | **57838.97** | **58442.25** | **58092.79** | **0.801** | **6.0** | **20** | **100** | **21** |

**Panel B. Focused K = 5 and K = 6 confirmation**

| **K** | **Initial best LL** | **Focused best LL** | **Retained AIC** | **Retained BIC** | **Successful starts** | **Within 0.01** | **Within 0.05** | **Within 0.10** |
| --- | --- | --- | --- | --- | --- | --- | --- | --- |
| 5 | -28930.817 | -28930.814 | 58029.63 | 58490.31 | 100 | 58 | 67 | 67 |
| 6 | -28809.492 | -28809.486 | 57838.97 | 58442.25 | 100 | 21 | 29 | 29 |

**Panel C. Retained K = 5 and K = 6 profile means**

| **K** | **Profile** | **AMO** | **EXT** | **INTJ** | **IDEN** | **INTR** |
| --- | --- | --- | --- | --- | --- | --- |
| 5 | P1 | 2.29 | 2.11 | 1.84 | 1.71 | 1.59 |
| 5 | P2 | 2.35 | 2.38 | 2.74 | 2.21 | 2.99 |
| 5 | P3 | 1.80 | 1.85 | 2.24 | 3.11 | 1.89 |
| 5 | P4 | 1.11 | 1.01 | 2.41 | 1.91 | 3.02 |
| 5 | P5 | 0.80 | 0.91 | 3.02 | 3.37 | 3.34 |
| 6 | P1 | 2.79 | 2.29 | 2.26 | 2.00 | 1.66 |
| 6 | P2 | 2.00 | 2.01 | 1.62 | 1.61 | 1.58 |
| 6 | P3 | 2.22 | 2.22 | 2.76 | 2.19 | 3.10 |
| 6 | P4 | 1.70 | 1.82 | 2.24 | 3.14 | 1.91 |
| 6 | P5 | 1.05 | 0.96 | 2.38 | 1.92 | 3.03 |
| 6 | P6 | 0.80 | 0.90 | 3.03 | 3.37 | 3.35 |

*Note.* K = number of profiles; LL = log-likelihood; AIC = Akaike information criterion; BIC = Bayesian information criterion; adjusted BIC = sample-size-adjusted BIC. The initial enumeration used one deterministic and 19 random starts for K = 2-6; K = 1 used one deterministic start. The focused confirmation used 100 new independent starts for K = 5 and K = 6. “Within” columns count focused starts whose log-likelihood was within the stated number of units of the best focused solution. The retained six-profile solution is bolded. Profile codes in Panel C are ordered by the internalization-ordering index within each K and are not intended as identical substantive labels across different K solutions.

**Table S8** *Final Six-Profile Parameter Estimates, Classification Quality, Transition Probabilities, and Posterior Expected Counts*

**Panel A. Profile-specific regulation means and 95% confidence intervals**

| **Profile** | **AMO [95% CI]** | **EXT [95% CI]** | **INTJ [95% CI]** | **IDEN [95% CI]** | **INTR [95% CI]** |
| --- | --- | --- | --- | --- | --- |
| P1, Amotivated-controlled | 2.79 [2.42, 3.16] | 2.29 [1.97, 2.62] | 2.26 [1.80, 2.71] | 2.00 [1.58, 2.41] | 1.66 [1.48, 1.83] |
| P2, Low-regulation | 2.00 [1.50, 2.50] | 2.01 [1.90, 2.11] | 1.62 [1.30, 1.93] | 1.61 [1.46, 1.76] | 1.58 [1.52, 1.64] |
| P3, Mixed intrinsic-controlled | 2.22 [2.08, 2.35] | 2.22 [2.04, 2.40] | 2.76 [2.63, 2.89] | 2.19 [2.09, 2.28] | 3.10 [2.98, 3.21] |
| P4, Identified-dominant | 1.70 [1.50, 1.91] | 1.82 [1.69, 1.94] | 2.24 [2.16, 2.32] | 3.14 [3.08, 3.21] | 1.91 [1.84, 1.98] |
| P5, Intrinsic-dominant | 1.05 [0.96, 1.13] | 0.96 [0.88, 1.05] | 2.38 [2.30, 2.47] | 1.92 [1.86, 1.98] | 3.03 [2.97, 3.09] |
| P6, High autonomous-introjected | 0.80 [0.76, 0.83] | 0.90 [0.87, 0.94] | 3.03 [2.99, 3.06] | 3.37 [3.34, 3.40] | 3.35 [3.32, 3.37] |

**Panel B. Posterior prevalence and classification quality by wave**

| **Profile** | **T1 prev %** | **T1 modal n** | **T1 avg posterior** | **T2 prev %** | **T2 modal n** | **T2 avg posterior** | **T3 prev %** | **T3 modal n** | **T3 avg posterior** |
| --- | --- | --- | --- | --- | --- | --- | --- | --- | --- |
| P1, Amotivated-controlled | 11.5 | 195 | 0.791 | 11.8 | 205 | 0.792 | 12.3 | 212 | 0.739 |
| P2, Low-regulation | 16.4 | 302 | 0.835 | 19.1 | 341 | 0.841 | 17.6 | 330 | 0.784 |
| P3, Mixed intrinsic-controlled | 6.2 | 97 | 0.813 | 6.0 | 102 | 0.788 | 7.1 | 117 | 0.750 |
| P4, Identified-dominant | 11.4 | 205 | 0.840 | 10.9 | 194 | 0.830 | 10.0 | 173 | 0.773 |
| P5, Intrinsic-dominant | 10.7 | 193 | 0.823 | 11.8 | 210 | 0.840 | 12.0 | 216 | 0.795 |
| P6, High autonomous-introjected | 43.8 | 788 | 0.953 | 40.4 | 728 | 0.949 | 41.0 | 732 | 0.926 |

**Panel C. T1 to T2 transition probabilities (%)**

| **Origin / destination** | **P1** | **P2** | **P3** | **P4** | **P5** | **P6** |
| --- | --- | --- | --- | --- | --- | --- |
| P1 | 85.0 | <0.1 | 2.5 | 7.2 | <0.1 | 5.3 |
| P2 | 0.7 | 73.1 | <0.1 | 2.0 | 6.2 | 18.0 |
| P3 | 7.2 | <0.1 | 75.3 | 1.3 | 8.0 | 8.1 |
| P4 | 4.2 | 15.5 | 1.8 | 70.3 | 2.7 | 5.5 |
| P5 | <0.1 | 12.4 | 1.4 | 2.3 | 73.4 | 10.5 |
| P6 | 2.2 | 9.1 | 1.6 | 3.2 | 4.8 | 79.1 |

**Panel D. T2 to T3 transition probabilities (%)**

| **Origin / destination** | **P1** | **P2** | **P3** | **P4** | **P5** | **P6** |
| --- | --- | --- | --- | --- | --- | --- |
| P1 | 74.9 | <0.1 | 5.9 | 9.0 | 2.7 | 7.4 |
| P2 | 0.2 | 68.6 | <0.1 | 5.6 | 5.6 | 20.1 |
| P3 | 15.3 | <0.1 | 76.7 | <0.1 | <0.1 | 7.9 |
| P4 | 10.8 | 15.9 | 1.9 | 55.6 | 0.9 | 14.9 |
| P5 | 4.7 | <0.1 | 2.3 | 5.5 | 73.4 | 14.1 |
| P6 | 1.9 | 7.0 | 3.1 | 2.9 | 4.5 | 80.5 |

**Panel E. T1 to T2 posterior expected transition counts**

| **Origin / destination** | **P1** | **P2** | **P3** | **P4** | **P5** | **P6** |
| --- | --- | --- | --- | --- | --- | --- |
| P1 | 173.8 | <0.1 | 5.2 | 14.7 | <0.1 | 10.7 |
| P2 | 2.1 | 213.6 | <0.1 | 5.8 | 18.0 | 52.6 |
| P3 | 8.0 | <0.1 | 82.7 | 1.5 | 8.8 | 8.9 |
| P4 | 8.5 | 31.4 | 3.6 | 143.0 | 5.5 | 11.3 |
| P5 | <0.1 | 23.7 | 2.7 | 4.4 | 140.1 | 20.0 |
| P6 | 17.3 | 70.7 | 12.7 | 25.2 | 37.2 | 616.2 |

**Panel F. T2 to T3 posterior expected transition counts**

| **Origin / destination** | **P1** | **P2** | **P3** | **P4** | **P5** | **P6** |
| --- | --- | --- | --- | --- | --- | --- |
| P1 | 157.1 | <0.1 | 12.4 | 18.9 | 5.7 | 15.6 |
| P2 | 0.6 | 232.7 | <0.1 | 18.9 | 19.1 | 68.1 |
| P3 | 16.4 | <0.1 | 82.1 | <0.1 | <0.1 | 8.5 |
| P4 | 21.1 | 30.9 | 3.7 | 108.2 | 1.7 | 29.0 |
| P5 | 9.9 | <0.1 | 4.7 | 11.5 | 153.9 | 29.6 |
| P6 | 13.8 | 50.2 | 22.6 | 21.0 | 32.5 | 579.6 |

*Note.* AMO = amotivation; EXT = external regulation; INTJ = introjected regulation; IDEN = identified regulation; INTR = intrinsic regulation; prev = posterior prevalence; avg posterior = average posterior probability among modal members. Profile means were constrained to be invariant across waves in the final model. Transition probabilities are row percentages conditional on the origin profile; values below 0.1% are shown as <0.1. Posterior expected counts are sums of posterior joint transition probabilities and can be fractional; values below 0.1 are shown as <0.1. Modal n is provided only as a classification-quality descriptor and was not treated as error-free profile membership in subsequent analyses.

**Table S9** *Complete Psychological-Need Predictor Results for All Eligible Pairwise Profile Transitions*

**Panel A. Primary need-satisfaction and need-frustration models**

| **Interval** | **Transition vs stability** | **Expected n** | **Need satisfaction OR [95% CI]** | **p** | **Holm p** | **Need frustration OR [95% CI]** | **p** | **Holm p** |
| --- | --- | --- | --- | --- | --- | --- | --- | --- |
| T1 to T2 | P1->P4 vs P1->P1 | 14.7 | 1.59 [1.11, 2.29] | .012 | .134 | 0.86 [0.59, 1.27] | .459 | 1.000 |
| T1 to T2 | P1->P6 vs P1->P1 | 10.7 | 1.10 [0.75, 1.60] | .636 | 1.000 | 0.76 [0.54, 1.06] | .102 | 1.000 |
| T1 to T2 | P2->P5 vs P2->P2 | 18.0 | 1.00 [0.59, 1.72] | .989 | 1.000 | **0.41 [0.27, 0.62]** | **<.001** | **<.001** |
| T1 to T2 | P2->P6 vs P2->P2 | 52.6 | 1.49 [1.02, 2.17] | .037 | .370 | 0.92 [0.68, 1.25] | .597 | 1.000 |
| T1 to T2 | P4->P2 vs P4->P4 | 31.4 | 0.71 [0.50, 1.03] | .071 | .641 | 0.92 [0.67, 1.28] | .638 | 1.000 |
| T1 to T2 | P4->P6 vs P4->P4 | 11.3 | 1.10 [0.67, 1.80] | .703 | 1.000 | 1.02 [0.59, 1.76] | .939 | 1.000 |
| T1 to T2 | P5->P2 vs P5->P5 | 23.7 | **0.47 [0.30, 0.76]** | **.002** | **.020** | 1.33 [0.84, 2.10] | .228 | 1.000 |
| T1 to T2 | P5->P6 vs P5->P5 | 20.0 | 1.00 [0.69, 1.44] | .987 | 1.000 | 1.63 [1.06, 2.50] | .027 | .292 |
| T1 to T2 | P6->P1 vs P6->P6 | 17.3 | 0.80 [0.49, 1.32] | .383 | 1.000 | 2.08 [1.22, 3.57] | .007 | .089 |
| T1 to T2 | P6->P2 vs P6->P6 | 70.7 | **0.49 [0.36, 0.67]** | **<.001** | **<.001** | 1.06 [0.74, 1.52] | .750 | 1.000 |
| T1 to T2 | P6->P3 vs P6->P6 | 12.7 | 0.98 [0.54, 1.77] | .938 | 1.000 | 1.45 [0.65, 3.21] | .363 | 1.000 |
| T1 to T2 | P6->P4 vs P6->P6 | 25.2 | 1.08 [0.65, 1.81] | .759 | 1.000 | 1.48 [0.87, 2.52] | .144 | 1.000 |
| T1 to T2 | P6->P5 vs P6->P6 | 37.2 | 0.84 [0.57, 1.23] | .372 | 1.000 | 1.00 [0.66, 1.53] | .988 | 1.000 |
| T2 to T3 | P1->P3 vs P1->P1 | 12.4 | 1.05 [0.67, 1.66] | .818 | 1.000 | 0.92 [0.57, 1.49] | .736 | 1.000 |
| T2 to T3 | P1->P4 vs P1->P1 | 18.9 | 1.03 [0.69, 1.54] | .885 | 1.000 | 0.58 [0.40, 0.85] | .005 | .085 |
| T2 to T3 | P1->P6 vs P1->P1 | 15.6 | 1.27 [0.79, 2.05] | .326 | 1.000 | 0.79 [0.52, 1.18] | .250 | 1.000 |
| T2 to T3 | P2->P4 vs P2->P2 | 18.9 | 1.15 [0.86, 1.54] | .353 | 1.000 | **0.62 [0.46, 0.84]** | **.002** | **.040** |
| T2 to T3 | P2->P5 vs P2->P2 | 19.1 | 1.11 [0.82, 1.51] | .502 | 1.000 | 0.72 [0.54, 0.98] | .037 | .486 |
| T2 to T3 | P2->P6 vs P2->P2 | 68.1 | 1.12 [0.85, 1.46] | .427 | 1.000 | 0.83 [0.63, 1.09] | .177 | 1.000 |
| T2 to T3 | P3->P1 vs P3->P3 | 16.4 | 1.07 [0.67, 1.69] | .788 | 1.000 | 1.71 [1.10, 2.64] | .016 | .229 |
| T2 to T3 | P4->P1 vs P4->P4 | 21.1 | 0.85 [0.63, 1.14] | .268 | 1.000 | 1.36 [1.02, 1.81] | .038 | .486 |
| T2 to T3 | P4->P2 vs P4->P4 | 30.9 | 0.71 [0.53, 0.97] | .030 | .485 | 1.14 [0.82, 1.58] | .435 | 1.000 |
| T2 to T3 | P4->P6 vs P4->P4 | 29.0 | 0.87 [0.58, 1.31] | .512 | 1.000 | 0.98 [0.67, 1.45] | .925 | 1.000 |
| T2 to T3 | P5->P4 vs P5->P5 | 11.5 | 1.00 [0.60, 1.68] | .997 | 1.000 | 1.48 [0.85, 2.59] | .170 | 1.000 |
| T2 to T3 | P5->P6 vs P5->P5 | 29.6 | 1.18 [0.86, 1.62] | .301 | 1.000 | 1.44 [0.93, 2.22] | .103 | 1.000 |
| T2 to T3 | P6->P1 vs P6->P6 | 13.8 | 1.36 [0.83, 2.24] | .221 | 1.000 | 1.77 [1.17, 2.67] | .007 | .104 |
| T2 to T3 | P6->P2 vs P6->P6 | 50.2 | 0.62 [0.44, 0.89] | .009 | .145 | 0.75 [0.49, 1.16] | .200 | 1.000 |
| T2 to T3 | P6->P3 vs P6->P6 | 22.6 | 1.28 [0.79, 2.05] | .312 | 1.000 | 1.34 [0.88, 2.04] | .169 | 1.000 |
| T2 to T3 | P6->P4 vs P6->P6 | 21.0 | 0.72 [0.49, 1.08] | .110 | 1.000 | 1.45 [0.92, 2.28] | .110 | 1.000 |
| T2 to T3 | P6->P5 vs P6->P6 | 32.5 | 0.81 [0.57, 1.15] | .236 | 1.000 | 1.02 [0.68, 1.51] | .934 | 1.000 |

**Panel B. Sensitivity models additionally controlling prior GLTEQ**

| **Interval** | **Transition vs stability** | **Expected n** | **Need satisfaction OR [95% CI]** | **p** | **Holm p** | **Need frustration OR [95% CI]** | **p** | **Holm p** |
| --- | --- | --- | --- | --- | --- | --- | --- | --- |
| T1 to T2 | P1->P4 vs P1->P1 | 14.7 | 1.71 [1.15, 2.55] | .008 | .089 | 0.81 [0.54, 1.22] | .318 | 1.000 |
| T1 to T2 | P1->P6 vs P1->P1 | 10.7 | 1.13 [0.76, 1.68] | .556 | 1.000 | 0.71 [0.51, 0.99] | .045 | .446 |
| T1 to T2 | P2->P5 vs P2->P2 | 18.0 | 1.00 [0.59, 1.68] | .989 | 1.000 | **0.41 [0.27, 0.64]** | **<.001** | **<.001** |
| T1 to T2 | P2->P6 vs P2->P2 | 52.6 | 1.51 [1.03, 2.21] | .033 | .334 | 0.91 [0.67, 1.24] | .563 | 1.000 |
| T1 to T2 | P4->P2 vs P4->P4 | 31.4 | 0.71 [0.49, 1.03] | .074 | .663 | 0.93 [0.67, 1.29] | .656 | 1.000 |
| T1 to T2 | P4->P6 vs P4->P4 | 11.3 | 1.06 [0.64, 1.74] | .830 | 1.000 | 1.04 [0.58, 1.87] | .884 | 1.000 |
| T1 to T2 | P5->P2 vs P5->P5 | 23.7 | **0.47 [0.30, 0.76]** | **.002** | **.021** | 1.34 [0.83, 2.15] | .230 | 1.000 |
| T1 to T2 | P5->P6 vs P5->P5 | 20.0 | 1.02 [0.71, 1.49] | .900 | 1.000 | 1.73 [1.09, 2.76] | .020 | .216 |
| T1 to T2 | P6->P1 vs P6->P6 | 17.3 | 0.78 [0.47, 1.29] | .326 | 1.000 | 2.13 [1.23, 3.68] | .007 | .083 |
| T1 to T2 | P6->P2 vs P6->P6 | 70.7 | **0.49 [0.36, 0.67]** | **<.001** | **<.001** | 1.06 [0.74, 1.54] | .737 | 1.000 |
| T1 to T2 | P6->P3 vs P6->P6 | 12.7 | 0.98 [0.56, 1.73] | .945 | 1.000 | 1.44 [0.64, 3.25] | .375 | 1.000 |
| T1 to T2 | P6->P4 vs P6->P6 | 25.2 | 1.13 [0.67, 1.92] | .647 | 1.000 | 1.44 [0.85, 2.46] | .178 | 1.000 |
| T1 to T2 | P6->P5 vs P6->P6 | 37.2 | 0.86 [0.58, 1.27] | .443 | 1.000 | 0.99 [0.64, 1.52] | .954 | 1.000 |
| T2 to T3 | P1->P3 vs P1->P1 | 12.4 | 1.06 [0.67, 1.68] | .791 | 1.000 | 0.91 [0.55, 1.50] | .711 | 1.000 |
| T2 to T3 | P1->P4 vs P1->P1 | 18.9 | 1.04 [0.67, 1.60] | .863 | 1.000 | 0.57 [0.39, 0.84] | .004 | .066 |
| T2 to T3 | P1->P6 vs P1->P1 | 15.6 | 1.33 [0.80, 2.22] | .276 | 1.000 | 0.73 [0.49, 1.09] | .127 | 1.000 |
| T2 to T3 | P2->P4 vs P2->P2 | 18.9 | 1.17 [0.87, 1.57] | .296 | 1.000 | **0.61 [0.45, 0.84]** | **.002** | **.035** |
| T2 to T3 | P2->P5 vs P2->P2 | 19.1 | 1.10 [0.80, 1.50] | .566 | 1.000 | 0.73 [0.54, 1.00] | .047 | .662 |
| T2 to T3 | P2->P6 vs P2->P2 | 68.1 | 1.13 [0.86, 1.48] | .389 | 1.000 | 0.82 [0.62, 1.08] | .160 | 1.000 |
| T2 to T3 | P3->P1 vs P3->P3 | 16.4 | 1.13 [0.70, 1.82] | .619 | 1.000 | 1.51 [0.98, 2.34] | .065 | .839 |
| T2 to T3 | P4->P1 vs P4->P4 | 21.1 | 0.86 [0.64, 1.16] | .329 | 1.000 | 1.33 [0.98, 1.81] | .065 | .839 |
| T2 to T3 | P4->P2 vs P4->P4 | 30.9 | 0.73 [0.53, 1.00] | .047 | .746 | 1.11 [0.79, 1.56] | .538 | 1.000 |
| T2 to T3 | P4->P6 vs P4->P4 | 29.0 | 0.85 [0.57, 1.28] | .433 | 1.000 | 1.00 [0.67, 1.50] | .999 | 1.000 |
| T2 to T3 | P5->P4 vs P5->P5 | 11.5 | 1.01 [0.59, 1.73] | .970 | 1.000 | 1.43 [0.80, 2.54] | .229 | 1.000 |
| T2 to T3 | P5->P6 vs P5->P5 | 29.6 | 1.17 [0.85, 1.60] | .334 | 1.000 | 1.46 [0.94, 2.28] | .094 | 1.000 |
| T2 to T3 | P6->P1 vs P6->P6 | 13.8 | 1.41 [0.86, 2.31] | .172 | 1.000 | 1.73 [1.15, 2.61] | .009 | .134 |
| T2 to T3 | P6->P2 vs P6->P6 | 50.2 | 0.63 [0.44, 0.89] | .010 | .162 | 0.75 [0.49, 1.16] | .191 | 1.000 |
| T2 to T3 | P6->P3 vs P6->P6 | 22.6 | 1.31 [0.80, 2.13] | .283 | 1.000 | 1.32 [0.87, 2.01] | .194 | 1.000 |
| T2 to T3 | P6->P4 vs P6->P6 | 21.0 | 0.74 [0.49, 1.10] | .139 | 1.000 | 1.43 [0.91, 2.26] | .124 | 1.000 |
| T2 to T3 | P6->P5 vs P6->P6 | 32.5 | 0.80 [0.56, 1.13] | .206 | 1.000 | 1.03 [0.69, 1.55] | .874 | 1.000 |

*Note.* Each transition was compared with stability in the same origin profile. Eligible comparisons required a posterior expected destination count of at least 10 and a posterior expected same-origin stability count of at least 50. ORs represent a one-pooled-standard-deviation increase in the predictor. Panel A includes need satisfaction and need frustration simultaneously. Panel B additionally controls origin-wave log(1 + weighted GLTEQ). P values were pooled across 50 multiply imputed data sets using Rubin's rules. Holm corrections were applied separately within interval, model, and predictor families; Holm-significant estimates are bolded. Expected n values are posterior expected counts and can be fractional. P1 = amotivated-controlled; P2 = low-regulation; P3 = mixed intrinsic-controlled; P4 = identified-dominant; P5 = intrinsic-dominant; P6 = high autonomous-introjected. All eligible models converged in all 50 imputations, HC3 robust covariance estimates were obtained, and no model errors were recorded.

**Table S10** *Complete Prospective Associations of T1-to-T2 Profile Transitions With T3 Leisure-Time Exercise and Sensitivity Analyses*

**Panel A. Primary model adjusted for T1 exercise**

| **Transition vs stability** | **Expected n** | **Adjusted T3 GLTEQ: stay [95% CI]** | **Adjusted T3 GLTEQ: move [95% CI]** | **Ratio (GLTEQ + 1) [95% CI]** | **p** | **Holm p** |
| --- | --- | --- | --- | --- | --- | --- |
| P1->P4 vs P1->P1 | 14.7 | 20.8 [18.6, 23.3] | 23.6 [19.2, 29.0] | 1.13 [0.91, 1.39] | .273 | .545 |
| P1->P6 vs P1->P1 | 10.7 | 20.9 [18.7, 23.4] | 36.6 [28.3, 47.2] | **1.72 [1.31, 2.25]** | **<.001** | **<.001** |
| P2->P5 vs P2->P2 | 18.0 | 19.1 [17.0, 21.5] | 25.0 [18.6, 33.5] | 1.29 [0.96, 1.74] | .092 | .277 |
| P2->P6 vs P2->P2 | 52.6 | 19.1 [17.0, 21.5] | 35.3 [29.4, 42.4] | **1.81 [1.47, 2.22]** | **<.001** | **<.001** |
| P4->P2 vs P4->P4 | 31.4 | 26.1 [23.0, 29.6] | 19.4 [15.8, 23.9] | 0.75 [0.61, 0.93] | .009 | .055 |
| P4->P6 vs P4->P4 | 11.3 | 26.2 [23.1, 29.7] | 35.5 [28.9, 43.5] | 1.34 [1.07, 1.68] | .010 | .055 |
| P5->P2 vs P5->P5 | 23.7 | 33.3 [29.8, 37.3] | 22.7 [17.9, 28.9] | **0.69 [0.54, 0.88]** | **.003** | **.021** |
| P5->P6 vs P5->P5 | 20.0 | 33.6 [30.0, 37.5] | 42.2 [35.0, 50.9] | 1.25 [1.02, 1.53] | .032 | .128 |
| P6->P1 vs P6->P6 | 17.3 | 41.3 [38.7, 43.9] | 20.8 [16.5, 26.2] | **0.52 [0.41, 0.65]** | **<.001** | **<.001** |
| P6->P2 vs P6->P6 | 70.7 | 41.2 [38.7, 43.9] | 22.7 [19.0, 27.2] | **0.56 [0.47, 0.67]** | **<.001** | **<.001** |
| P6->P3 vs P6->P6 | 12.7 | 41.2 [38.7, 43.9] | 25.5 [18.4, 35.3] | **0.63 [0.46, 0.86]** | **.004** | **.028** |
| P6->P4 vs P6->P6 | 25.2 | 41.2 [38.7, 43.8] | 24.7 [18.5, 32.9] | **0.61 [0.46, 0.81]** | **<.001** | **.005** |
| P6->P5 vs P6->P6 | 37.2 | 41.2 [38.7, 43.9] | 37.9 [32.6, 44.0] | 0.92 [0.79, 1.07] | .295 | .545 |

**Panel B. Stricter T1 + T2 exercise adjustment and raw-outcome sensitivity**

| **Transition vs stability** | **T1 + T2 adjusted ratio [95% CI]** | **p** | **Holm p** | **Raw GLTEQ difference [95% CI]** | **p** | **Holm p** | **Same direction all specs** |
| --- | --- | --- | --- | --- | --- | --- | --- |
| P1->P4 vs P1->P1 | 1.07 [0.89, 1.28] | .486 | .971 | 2.6 [-2.4, 7.7] | .309 | .309 | Yes |
| P1->P6 vs P1->P1 | 1.33 [1.03, 1.73] | .031 | .309 | **17.6 [9.0, 26.3]** | **<.001** | **<.001** | Yes |
| P2->P5 vs P2->P2 | 1.17 [0.87, 1.57] | .306 | .918 | 7.8 [0.2, 15.4] | .044 | .198 | Yes |
| P2->P6 vs P2->P2 | **1.48 [1.22, 1.80]** | **<.001** | **<.001** | **20.7 [11.1, 30.3]** | **<.001** | **<.001** | Yes |
| P4->P2 vs P4->P4 | 0.86 [0.71, 1.03] | .108 | .650 | -7.0 [-13.3, -0.6] | .033 | .198 | Yes |
| P4->P6 vs P4->P4 | 1.18 [0.95, 1.45] | .127 | .650 | 8.5 [0.1, 17.0] | .048 | .198 | Yes |
| P5->P2 vs P5->P5 | 0.81 [0.64, 1.03] | .081 | .569 | **-11.7 [-19.0, -4.4]** | **.002** | **.013** | Yes |
| P5->P6 vs P5->P5 | 1.16 [0.95, 1.41] | .143 | .650 | 12.4 [0.5, 24.4] | .042 | .198 | Yes |
| P6->P1 vs P6->P6 | 0.78 [0.63, 0.97] | .024 | .260 | **-29.0 [-38.0, -20.0]** | **<.001** | **<.001** | Yes |
| P6->P2 vs P6->P6 | 0.86 [0.74, 1.01] | .062 | .500 | **-26.1 [-32.9, -19.3]** | **<.001** | **<.001** | Yes |
| P6->P3 vs P6->P6 | 0.71 [0.53, 0.95] | .020 | .244 | **-18.9 [-29.7, -8.1]** | **<.001** | **.005** | Yes |
| P6->P4 vs P6->P6 | 0.74 [0.57, 0.98] | .033 | .309 | **-17.0 [-26.3, -7.7]** | **<.001** | **.003** | Yes |
| P6->P5 vs P6->P6 | 1.00 [0.87, 1.16] | .983 | .983 | -7.5 [-14.5, -0.6] | .034 | .198 | No |

*Note.* Only T1-to-T2 transitions were used to preserve temporal ordering relative to T3 exercise. Each off-diagonal transition was compared with stability in the same origin profile; eligible transitions required a posterior expected destination count of at least 10 and a posterior expected same-origin stability count of at least 50. Panel A models log(1 + T3 GLTEQ) and controls log(1 + T1 GLTEQ); adjusted means are back-transformed to the weighted GLTEQ scale. The ratio is exp(B) and compares GLTEQ + 1 for movers versus stayers. Panel B first adds log(1 + T2 GLTEQ), then reports a raw-score sensitivity model controlling raw T1 GLTEQ. Holm corrections were applied across eligible transitions within each specification; Holm-significant estimates are bolded. P1 = amotivated-controlled; P2 = low-regulation; P3 = mixed intrinsic-controlled; P4 = identified-dominant; P5 = intrinsic-dominant; P6 = high autonomous-introjected. All models were completed in all 50 imputations with participant-clustered HC1 covariance estimates, and no model errors were recorded.

**Supplementary Figure S1. Complete associations of exercise-specific need satisfaction and need frustration with eligible pairwise profile transitions.** Forest plots show odds ratios and 95% confidence intervals for all eligible pairwise profile-transition comparisons. Each comparison contrasts the selected destination transition with stability in the same origin profile. Primary models entered origin-wave need satisfaction and need frustration simultaneously. Sensitivity models additionally controlled origin-wave log(1 + GLTEQ). Odds ratios represent a one pooled-standard-deviation increase in the focal predictor. Posterior expected transition counts are shown in brackets and can be fractional. P1 = amotivated–controlled; P2 = low-regulation; P3 = mixed intrinsic–controlled; P4 = identified-dominant; P5 = intrinsic-dominant; P6 = high autonomous–introjected.

**Supplementary Figure S2. Raw-scale differences in T3 leisure-time exercise associated with T1-to-T2 exercise-regulation profile transitions.** Points and 95% confidence intervals represent adjusted differences in the raw weighted T3 GLTEQ score between students probabilistically represented by each T1-to-T2 transition and students remaining in the same origin profile, controlling the raw T1 GLTEQ score. Positive values indicate higher T3 exercise among movers; negative values indicate lower T3 exercise. Filled points identify contrasts significant after Holm correction. Posterior expected transition counts are shown in brackets and can be fractional. P1 = amotivated–controlled; P2 = low-regulation; P3 = mixed intrinsic–controlled; P4 = identified-dominant; P5 = intrinsic-dominant; P6 = high autonomous–introjected.

**Supplementary Figure S3. Bayesian information criterion trajectories across evaluated profile solutions.** Panel A displays BIC values for the one- through six-profile longitudinal latent transition models. Panels B–D display BIC values for the one- through six-profile wave-specific latent profile models under equal diagonal (EEI) and equal unrestricted (EEE) covariance structures at T1, T2, and T3, respectively. Lower BIC values indicate better relative fit after penalizing model complexity. The plots are provided as visual aids to the multi-criterion profile-enumeration process; profile selection also considered entropy, minimum profile size, random-start reproducibility, substantive distinctiveness, and consistency across wave-specific and longitudinal solutions.
